# Supplementary material for: Prevotella copri alleviates hyperglycemia and regulates gut microbiota and metabolic profiles in mice
Source: mSystems. 2024 Jun 27;9(7):e00532-24. doi: 10.1128/msystems.00532-24 (PMC11265406; doi:10.1128/msystems.00532-24)
Supplement: Supplemental material — Legends for Fig. S1-S4; Tables S1-S4. [file msystems.00532-24-s0005.docx]

**Figure S1. Phylogenetic analysis of *P. copri*.** (A) Workflow of data collection. (B) Phylogenetic tree of 184 genomes of *P. copri* based on the concatenation of 134 core genes by Roary, *Prevotella melaninogenica* ATCC 25845^T^ was used as an outgroup. The five different clades are distinguished by five different color blocks, the strains isolated in this study were marked with red stars. (C) Heatmap of ANI values of *P. copri* complex based on the 184 whole genome sequences. (D) The ANI value within a clade and between clades of *P. copri* complex. (E) The unrooted phylogenetic tree of a set of 928 core genes was obtained by Roary using 158 genomes from clade A. The six different sub-branches are denoted by distinct colors. The strains isolated in this study were labeled with red stars. (F) Heatmap of ANI values of *P. copri* complex based on 158 genomes from clade A.

**Figure S2. Effect of *P. copri* on glucose consumption.** The strains were classified into clade A1 (red columns), clade A2 (green columns) and clade C (blue columns) based on taxonomic position. Data are expressed as mean ± SD values.

**Figure S3. Effect of *P. copri* on serum total cholesterol (TC), serum triglycerides (TG), high-density lipoprotein (HDL-C) and low-density lipoprotein (LDL-C) in db/db mice (n=6).** * p<0.05, ** p<0.01, *** p<0.001.

**Figure S4 Differential distribution of functional gene categories in *P. copri* HF2130, DSM 18205, HF2123 and HF1478. (A) Comparsion of four strains of *P. copri*. (B) Information of the CAZy_family unique to the HF2130 genome. (C) COG categories present in HF2130 only.**

**Table S1. Sequence of primers used in this study.**

| Target | Forward and reverse primers |
| --- | --- |
| Prevotella copri | F: CACRGTAAACGATGGATGCC |
|  | R: GGTCGGGTTGCAGACC |
| GLP-1 | F: TCTCTAGGCTGCCGACTGGT |
|  | R: CTCCGAGAACACCGAGAAGG |
| GCG | F: ATTCAT TGCTTGGCTGGTGA |
|  | R: CCAGAATGGTGCTCATCTCG |
| PC1/3 | F: ACATGGGGAGAGAATCCTGTAGGCA |
|  | R: CATGGCCTTTGAAGGAGTTCCTTGT |
| β-actin | F: GGTCATCACTATTGGCAACG |
|  | R: ACGGATGTCAACGTCACACT |

**Table S2 Genomic information of the strains used in this study.**

| No. | Strain | BioSample | Assembly | Level | Size(Mb) | GC% | tRNA | rRNA | other RNA | Pseudogene | WGS | location | Scaffolds | CDS | Genes | Host |
| --- | --- | --- | --- | --- | --- | --- | --- | --- | --- | --- | --- | --- | --- | --- | --- | --- |
| 1 | AF10-17 | SAMN09734200 | GCA_003465495.1 | Scaffold | 3.69251 | 44.9 | 66 | 5 | 2 | 85 | QSAV01 | China:Shenzhen | 146 | 2888 | 3046 | Homo sapiens |
| 2 | AF11-14 | SAMN09734207 | GCA_003465445.1 | Scaffold | 3.83605 | 44.9 | 71 | 4 | 2 | 105 | QSAQ01 | China:Shenzhen | 125 | 3137 | 3319 | Homo sapiens |
| 3 | AF12-50 | SAMN09734223 | GCA_003464975.1 | Scaffold | 3.91281 | 45 | 59 | 7 | 2 | 105 | QSAG01 | China:Shenzhen | 120 | 3209 | 3382 | Homo sapiens |
| 4 | AF15-25 | SAMN09734282 | GCA_003465215.1 | Scaffold | 3.78486 | 44.7 | 70 | 3 | 2 | 89 | QRYP01 | China:Shenzhen | 144 | 3066 | 3230 | Homo sapiens |
| 5 | AF22-1 | SAMN09734437 | GCA_003459305.1 | Contig | 3.80918 | 44.8 | 59 | 19 | 2 | 456 | QRVN01 | China:Shenzhen | 139 | 2769 | 3305 | Homo sapiens |
| 6 | AF24-12 | SAMN09734458 | GCA_003459205.1 | Scaffold | 3.869 | 44.6 | 66 | 4 | 2 | 104 | QRVA01 | China:Shenzhen | 150 | 3097 | 3273 | Homo sapiens |
| 7 | AF29-19 | SAMN09734557 | GCA_003457655.1 | Scaffold | 3.69303 | 45 | 52 | 5 | 2 | 95 | QRSU01 | China:Shenzhen | 122 | 2881 | 3035 | Homo sapiens |
| 8 | AF38-11 | SAMN09734673 | GCA_003474655.1 | Scaffold | 3.68604 | 45 | 69 | 5 | 2 | 80 | QROP01 | China:Shenzhen | 114 | 2882 | 3038 | Homo sapiens |
| 9 | AF43-2 | SAMN09734711 | GCA_003472885.1 | Contig | 3.76236 | 45 | 48 | 25 | 2 | 492 | QRNN01 | China:Shenzhen | 212 | 2694 | 3261 | Homo sapiens |
| 10 | AF46-2NS | SAMN09734726 | GCA_003473935.1 | Contig | 3.44599 | 45 | 51 | 18 | 2 | 591 | QRNB01 | China:Shenzhen | 209 | 2320 | 2982 | Homo sapiens |
| 11 | AM16-54 | SAMN09734839 | GCA_003471795.1 | Scaffold | 3.86211 | 44.9 | 62 | 4 | 2 | 94 | QRKB01 | China:Shenzhen | 150 | 3157 | 3319 | Homo sapiens |
| 12 | AM22-1 | SAMN09734897 | GCA_003471195.1 | Scaffold | 3.75623 | 44.9 | 75 | 6 | 2 | 105 | QRIN01 | China:Shenzhen | 185 | 3062 | 3250 | Homo sapiens |
| 13 | AM22-19 | SAMN09734909 | GCA_003471085.1 | Scaffold | 3.73369 | 44.9 | 72 | 6 | 2 | 94 | QRIF01 | China:Shenzhen | 197 | 3046 | 3220 | Homo sapiens |
| 14 | AM22-2 | SAMN09734910 | GCA_003471065.1 | Scaffold | 3.81477 | 44.9 | 77 | 9 | 2 | 115 | QRIE01 | China:Shenzhen | 182 | 3126 | 3329 | Homo sapiens |
| 15 | AM42-23AC | SAMN09736550 | GCA_003467935.1 | Scaffold | 3.98603 | 44.6 | 72 | 4 | 2 | 103 | QSFW01 | China:Shenzhen | 132 | 3315 | 3496 | Homo sapiens |
| 16 | An893 | SAMN15872560 | GCA_021531415.1 | Contig | 3.36782 | 44.9 | 49 | 7 | 2 | 77 | JADYTV01 | Czech Republic | 170 | 2733 | 2868 | Sus scrofa |
| 17 | BU4165 | SAMN12583292 | GCA_009494485.1 | Contig | 3.70079 | 45.1 | 47 | 7 | 2 | 84 | VZBX01 | USA: New York | 139 | 2911 | 3051 | Homo sapiens |
| 18 | BU41712 | SAMN12583297 | GCA_009494455.1 | Contig | 3.77095 | 44.8 | 50 | 5 | 2 | 91 | VZBZ01 | USA: New York | 183 | 3130 | 3278 | Homo sapiens |
| 19 | BU41813 | SAMN12583298 | GCA_009494345.1 | Contig | 3.78439 | 45 | 51 | 7 | 2 | 84 | VZCA01 | USA: New York | 134 | 3032 | 3176 | Homo sapiens |
| 20 | BVe4116 | SAMN12583253 | GCA_009495255.1 | Contig | 3.56939 | 45.1 | 61 | 4 | 2 | 63 | VZAK01 | USA: New York | 94 | 2889 | 3019 | Homo sapiens |
| 21 | BVe41210 | SAMN12583295 | GCA_009494435.1 | Contig | 3.56395 | 45.1 | 60 | 3 | 2 | 65 | VZBY01 | USA: New York | 77 | 2877 | 3007 | Homo sapiens |
| 22 | BVe41219 | SAMN12583268 | GCA_009494955.1 | Contig | 3.56439 | 45.1 | 58 | 3 | 2 | 73 | VZAZ01 | USA: New York | 75 | 2873 | 3009 | Homo sapiens |
| 23 | DSM 18205 | SAMN00008847 | GCA_000157935.1 | Scaffold | 3.51247 | 44.8 | 59 | 16 | 2 | 75 | ACBX02 | JAPAN | 27 | 2813 | 2965 | Homo sapiens |
| 24 | HDA03 | SAMN19158392 | GCA_019249855.1 | Contig | 3.74714 | 44.8 | 56 | 13 | 2 | 80 | JAHRGA01 | Germany:Braunschweig | 101 | 2917 | 3068 | Homo sapiens |
| 25 | HDA04 | SAMN19158393 | GCA_019249865.1 | Contig | 3.72155 | 44.8 | 58 | 15 | 2 | 68 | JAHRGB01 | Germany:Braunschweig | 109 | 2904 | 3047 | Homo sapiens |
| 26 | HDB01 | SAMN19158394 | GCA_019249825.1 | Contig | 3.67753 | 44.9 | 69 | 10 | 2 | 49 | JAHRGC01 | Germany:Braunschweig | 29 | 2932 | 3062 | Homo sapiens |
| 27 | HDC01 | SAMN19158395 | GCA_019249795.1 | Contig | 3.5852 | 45 | 66 | 8 | 2 | 83 | JAHRGD01 | Germany:Braunschweig | 99 | 2808 | 2967 | Homo sapiens |
| 28 | HDD04 | SAMN19158396 | GCA_019249765.1 | Contig | 3.8884 | 44.9 | 71 | 13 | 2 | 65 | JAHRGE01 | Germany:Braunschweig | 113 | 3112 | 3263 | Homo sapiens |
| 29 | HDD05 | SAMN19158397 | GCA_019249755.1 | Contig | 3.97541 | 44.8 | 65 | 13 | 2 | 68 | JAHRGF01 | Germany:Braunschweig | 109 | 3171 | 3319 | Homo sapiens |
| 30 | HDE04 | SAMN19158399 | GCA_019249805.1 | Contig | 4.21273 | 45.3 | 68 | 15 | 2 | 117 | JAHRGH01 | Germany:Braunschweig | 201 | 3422 | 3624 | Homo sapiens |
| 31 | HDE06 | SAMN19158400 | GCA_019249655.1 | Contig | 4.11995 | 46.2 | 79 | 19 | 2 | 95 | JAHRGI01 | Germany:Braunschweig | 155 | 3140 | 3335 | Homo sapiens |
| 32 | HF123 | SAMN29625245 | GCA_024330525.1 | Contig | 3.68538 | 44.9 | 57 | 18 | 2 | 55 | JANDWI01 | China:Beijing | 66 | 2954 | 3094 | Homo sapiens |
| 33 | HF129 | SAMN29625246 | GCA_024330985.1 | Contig | 3.79414 | 44.8 | 63 | 19 | 2 | 57 | JANDWJ01 | China:Beijing | 83 | 3143 | 3294 | Homo sapiens |
| 34 | HF1463 | SAMN29625247 | GCA_024330865.1 | Contig | 3.67933 | 44.9 | 66 | 17 | 2 | 45 | JANDWK01 | China:Beijing | 104 | 2922 | 3061 | Homo sapiens |
| 35 | HF1470 | SAMN29625248 | GCA_024330565.1 | Contig | 3.68099 | 44.9 | 66 | 16 | 2 | 43 | JANDWL01 | China:Beijing | 106 | 2930 | 3067 | Homo sapiens |
| 36 | HF1474 | SAMN29625249 | GCA_024330625.1 | Contig | 3.67808 | 44.9 | 66 | 16 | 2 | 47 | JANDWM01 | China:Beijing | 109 | 2922 | 3064 | Homo sapiens |
| 37 | HF1476 | SAMN29625250 | GCA_024330605.1 | Contig | 3.68029 | 44.9 | 66 | 16 | 2 | 47 | JANDWN01 | China:Beijing | 104 | 2923 | 3063 | Homo sapiens |
| 38 | HF1478 | SAMN29625251 | GCA_024330575.1 | Contig | 3.67912 | 44.9 | 66 | 16 | 2 | 47 | JANDWO01 | China:Beijing | 109 | 2924 | 3066 | Homo sapiens |
| 39 | HF1479 | SAMN29625252 | GCA_024330465.1 | Contig | 3.57546 | 44.9 | 62 | 16 | 2 | 46 | JANDWP01 | China:Beijing | 103 | 2833 | 2967 | Homo sapiens |
| 40 | HF1481 | SAMN29625253 | GCA_024329915.1 | Contig | 3.6807 | 44.9 | 66 | 16 | 2 | 44 | JANDWQ01 | China:Beijing | 109 | 2928 | 3067 | Homo sapiens |
| 41 | HF1482 | SAMN29625254 | GCA_024330385.1 | Contig | 3.75201 | 44.9 | 66 | 16 | 2 | 47 | JANDWR01 | China:Beijing | 110 | 2998 | 3140 | Homo sapiens |
| 42 | HF1484 | SAMN29625255 | GCA_024330415.1 | Contig | 3.65897 | 44.9 | 66 | 14 | 2 | 39 | JANDWS01 | China:Beijing | 145 | 2918 | 3050 | Homo sapiens |
| 43 | HF1500 | SAMN29625256 | GCA_024330785.1 | Contig | 3.68071 | 44.9 | 66 | 16 | 2 | 46 | JANDWT01 | China:Beijing | 106 | 2927 | 3063 | Homo sapiens |
| 44 | HF1805 | SAMN29625257 | GCA_024330505.1 | Contig | 3.91032 | 44.6 | 62 | 16 | 2 | 53 | JANDWU01 | China:Beijing | 124 | 3181 | 3328 | Homo sapiens |
| 45 | HF1816 | SAMN29625258 | GCA_024330545.1 | Contig | 3.91084 | 44.6 | 61 | 15 | 2 | 50 | JANDWV01 | China:Beijing | 130 | 3194 | 3333 | Homo sapiens |
| 46 | HF1822 | SAMN29625259 | GCA_024330405.1 | Contig | 3.85476 | 44.7 | 62 | 15 | 2 | 56 | JANDWW01 | China:Beijing | 157 | 3130 | 3277 | Homo sapiens |
| 47 | HF1825 | SAMN29625260 | GCA_024330485.1 | Contig | 3.91161 | 44.6 | 62 | 18 | 2 | 54 | JANDWX01 | China:Beijing | 122 | 3180 | 3330 | Homo sapiens |
| 48 | HF2100 | SAMN29625261 | GCA_024330435.1 | Contig | 3.5975 | 45.8 | 68 | 19 | 2 | 50 | JANDWY01 | China:Beijing | 88 | 2850 | 3008 | Homo sapiens |
| 49 | HF2106 | SAMN24371668 | GCA_021344195.1 | Contig | 3.77902 | 44.8 | 58 | 20 | 2 | 50 | JAJTVO01 | China:Beijing | 66 | 2996 | 3126 | Homo sapiens |
| 50 | HF2107 | SAMN29625262 | GCA_024330365.1 | Contig | 3.65409 | 45.7 | 71 | 21 | 2 | 51 | JANDWZ01 | China:Beijing | 85 | 2895 | 3049 | Homo sapiens |
| 51 | HF2110 | SAMN24424716 | GCA_021440055.1 | Contig | 3.68658 | 44.9 | 57 | 17 | 2 | 59 | JAJTTD01 | China:Beijing | 67 | 2952 | 3087 | Homo sapiens |
| 52 | HF2112 | SAMN29625263 | GCA_024330295.1 | Contig | 3.60253 | 45.8 | 70 | 21 | 2 | 51 | JANDXA01 | China:Beijing | 82 | 2854 | 3013 | Homo sapiens |
| 53 | HF2117 | SAMN29625264 | GCA_024330875.1 | Contig | 3.67578 | 45.8 | 70 | 19 | 2 | 48 | JANDXB01 | China:Beijing | 86 | 2945 | 3091 | Homo sapiens |
| 54 | HF2119 | SAMN29625265 | GCA_024330905.1 | Contig | 3.60996 | 45.8 | 70 | 21 | 2 | 50 | JANDXC01 | China:Beijing | 89 | 2863 | 3017 | Homo sapiens |
| 55 | HF2123 | SAMN29625266 | GCA_024329905.1 | Contig | 3.67372 | 44.9 | 57 | 18 | 2 | 52 | JANDXD01 | China:Beijing | 55 | 2938 | 3081 | Homo sapiens |
| 56 | HF2128 | SAMN29625267 | GCA_024330665.1 | Contig | 3.66367 | 45.8 | 71 | 20 | 3 | 50 | JANDXE01 | China:Beijing | 83 | 2905 | 3061 | Homo sapiens |
| 57 | HF2129 | SAMN29625268 | GCA_024330005.1 | Contig | 3.67435 | 44.9 | 57 | 17 | 2 | 55 | JANDXF01 | China:Beijing | 52 | 2936 | 3076 | Homo sapiens |
| 58 | HF2130 | SAMN29625269 | GCA_024329925.1 | Contig | 3.61518 | 45.8 | 74 | 20 | 2 | 48 | JANDXG01 | China:Beijing | 85 | 2871 | 3027 | Homo sapiens |
| 59 | HF288 | SAMN29625270 | GCA_024329865.1 | Contig | 3.84298 | 44.8 | 65 | 18 | 2 | 56 | JANDXH01 | China:Beijing | 83 | 3167 | 3325 | Homo sapiens |
| 60 | HF29 | SAMN29625271 | GCA_024330795.1 | Contig | 3.77835 | 44.8 | 59 | 22 | 2 | 46 | JANDXI01 | China:Beijing | 62 | 3000 | 3144 | Homo sapiens |
| 61 | HF298 | SAMN29625272 | GCA_024330725.1 | Contig | 3.77081 | 44.8 | 58 | 19 | 2 | 54 | JANDXJ01 | China:Beijing | 66 | 3079 | 3221 | Homo sapiens |
| 62 | HF299 | SAMN29625273 | GCA_024330745.1 | Contig | 3.76655 | 44.8 | 58 | 19 | 2 | 55 | JANDXK01 | China:Beijing | 74 | 3066 | 3214 | Homo sapiens |
| 63 | HF32 | SAMN29625274 | GCA_024330735.1 | Contig | 3.77643 | 44.8 | 56 | 19 | 2 | 46 | JANDXL01 | China:Beijing | 68 | 3003 | 3139 | Homo sapiens |
| 64 | HF40 | SAMN29625275 | GCA_024330685.1 | Contig | 3.68771 | 44.9 | 57 | 17 | 2 | 57 | JANDXM01 | China:Beijing | 64 | 2961 | 3104 | Homo sapiens |
| 65 | HF41 | SAMN29625276 | GCA_024330705.1 | Contig | 3.75834 | 44.8 | 56 | 17 | 2 | 53 | JANDXN01 | China:Beijing | 69 | 3065 | 3208 | Homo sapiens |
| 66 | HF43 | SAMN29625277 | GCA_024330845.1 | Contig | 3.68462 | 44.9 | 57 | 18 | 2 | 54 | JANDXO01 | China:Beijing | 65 | 2958 | 3100 | Homo sapiens |
| 67 | HF44 | SAMN29625278 | GCA_024330825.1 | Contig | 3.69369 | 44.9 | 59 | 18 | 2 | 57 | JANDXP01 | China:Beijing | 53 | 2969 | 3113 | Homo sapiens |
| 68 | HF52 | SAMN29625279 | GCA_024331205.1 | Contig | 3.77771 | 44.8 | 58 | 22 | 2 | 46 | JANDXQ01 | China:Beijing | 64 | 3003 | 3145 | Homo sapiens |
| 69 | HF88 | SAMN29625280 | GCA_024331165.1 | Contig | 3.68509 | 44.9 | 59 | 20 | 2 | 54 | JANDXR01 | China:Beijing | 60 | 2954 | 3099 | Homo sapiens |
| 70 | HF89 | SAMN29625281 | GCA_024330645.1 | Contig | 3.71237 | 44.8 | 58 | 20 | 2 | 55 | JANDXS01 | China:Beijing | 70 | 3016 | 3163 | Homo sapiens |
| 71 | iA622 | SAMN12583299 | GCA_009494395.1 | Contig | 3.93035 | 45.2 | 50 | 4 | 2 | 93 | VZCB01 | USA: New York | 112 | 3059 | 3208 | Homo sapiens |
| 72 | iA624 | SAMN12583283 | GCA_009494655.1 | Contig | 3.98303 | 45.7 | 60 | 7 | 2 | 80 | VZBP01 | USA: New York | 214 | 3140 | 3289 | Homo sapiens |
| 73 | iAA108 | SAMN12583300 | GCA_009494365.1 | Contig | 3.81928 | 44.7 | 44 | 5 | 2 | 56 | VZCC01 | USA: New York | 111 | 3063 | 3170 | Homo sapiens |
| 74 | iAA615 | SAMN12583301 | GCA_009494375.1 | Contig | 3.90842 | 45.4 | 49 | 7 | 2 | 78 | VZCD01 | USA: New York | 170 | 3106 | 3242 | Homo sapiens |
| 75 | iAA917 | SAMN12583250 | GCA_009495335.1 | Contig | 3.9057 | 45.5 | 55 | 8 | 2 | 72 | VZAH01 | USA: New York | 167 | 3098 | 3235 | Homo sapiens |
| 76 | iAK1212 | SAMN12583302 | GCA_009494335.1 | Contig | 3.5017 | 44.9 | 43 | 7 | 2 | 76 | VZCE01 | USA: New York | 96 | 2767 | 2895 | Homo sapiens |
| 77 | iAK1213 | SAMN12583303 | GCA_009494305.1 | Contig | 3.49303 | 44.9 | 45 | 6 | 2 | 72 | VZCF01 | USA: New York | 116 | 2761 | 2886 | Homo sapiens |
| 78 | iAK1214 | SAMN12583304 | GCA_009494275.1 | Contig | 3.69827 | 44.9 | 54 | 9 | 2 | 73 | VZCG01 | USA: New York | 105 | 2925 | 3063 | Homo sapiens |
| 79 | iAK1218 | SAMN12583305 | GCA_009494285.1 | Contig | 3.72665 | 44.9 | 54 | 4 | 2 | 76 | VZCH01 | USA: New York | 116 | 2976 | 3112 | Homo sapiens |
| 80 | iAK1219 | SAMN12583282 | GCA_009494675.1 | Contig | 3.68797 | 44.9 | 55 | 4 | 2 | 86 | VZBO01 | USA: New York | 110 | 2919 | 3066 | Homo sapiens |
| 81 | iAK127 | SAMN12583277 | GCA_009494745.1 | Contig | 3.49406 | 44.9 | 46 | 6 | 2 | 73 | VZBI01 | USA: New York | 118 | 2774 | 2901 | Homo sapiens |
| 82 | iAK128 | SAMN12583306 | GCA_009494245.1 | Contig | 3.41183 | 45 | 49 | 9 | 2 | 74 | VZCI01 | USA: New York | 92 | 2680 | 2814 | Homo sapiens |
| 83 | iAK261 | SAMN12583280 | GCA_009494635.1 | Contig | 3.45677 | 44.9 | 52 | 8 | 2 | 65 | VZBL01 | USA: New York | 101 | 2741 | 2868 | Homo sapiens |
| 84 | iAK2612 | SAMN12583270 | GCA_009494885.1 | Contig | 3.46885 | 44.9 | 47 | 7 | 2 | 67 | VZBB01 | USA: New York | 120 | 2753 | 2876 | Homo sapiens |
| 85 | iAK2615 | SAMN12583267 | GCA_009494975.1 | Contig | 3.48024 | 44.9 | 45 | 8 | 2 | 71 | VZAY01 | USA: New York | 95 | 2746 | 2872 | Homo sapiens |
| 86 | iAK2616 | SAMN12583307 | GCA_009494235.1 | Contig | 3.47635 | 44.9 | 47 | 7 | 2 | 64 | VZCJ01 | USA: New York | 100 | 2772 | 2892 | Homo sapiens |
| 87 | iAK2617 | SAMN12583308 | GCA_009494715.1 | Contig | 3.69488 | 44.9 | 59 | 5 | 2 | 80 | VZBM01 | USA: New York | 108 | 2927 | 3073 | Homo sapiens |
| 88 | iAK2620 | SAMN12583289 | GCA_009494535.1 | Contig | 3.46958 | 44.9 | 49 | 8 | 2 | 75 | VZBU01 | USA: New York | 109 | 2757 | 2891 | Homo sapiens |
| 89 | iAK263 | SAMN12583259 | GCA_009495135.1 | Contig | 3.47059 | 45 | 52 | 7 | 2 | 71 | VZAQ01 | USA: New York | 113 | 2750 | 2882 | Homo sapiens |
| 90 | iAK264 | SAMN12583269 | GCA_009494935.1 | Contig | 3.49243 | 44.9 | 48 | 5 | 2 | 70 | VZBA01 | USA: New York | 115 | 2782 | 2907 | Homo sapiens |
| 91 | iAK2651 | SAMN12583278 | GCA_009494795.1 | Contig | 3.6895 | 44.9 | 55 | 4 | 2 | 80 | VZBJ01 | USA: New York | 123 | 2945 | 3086 | Homo sapiens |
| 92 | iAK266 | SAMN12583258 | GCA_009495265.1 | Contig | 3.47591 | 44.9 | 53 | 9 | 2 | 81 | VZAJ01 | USA: New York | 121 | 2752 | 2897 | Homo sapiens |
| 93 | iAK268 | SAMN12583256 | GCA_009495205.1 | Contig | 3.67025 | 44.9 | 59 | 3 | 2 | 153 | VZAO01 | USA: New York | 236 | 2933 | 3150 | Homo sapiens |
| 94 | iAK269 | SAMN12583287 | GCA_009494555.1 | Contig | 3.47516 | 44.9 | 48 | 9 | 2 | 69 | VZBS01 | USA: New York | 100 | 2744 | 2872 | Homo sapiens |
| 95 | iAK2711 | SAMN12583309 | GCA_009494195.1 | Contig | 3.68513 | 44.9 | 56 | 9 | 2 | 76 | VZCK01 | USA: New York | 118 | 2934 | 3077 | Homo sapiens |
| 96 | iAK2712 | SAMN12583290 | GCA_009494475.1 | Contig | 3.45564 | 44.9 | 51 | 9 | 2 | 65 | VZBV01 | USA: New York | 103 | 2743 | 2870 | Homo sapiens |
| 97 | iAK2713 | SAMN12583275 | GCA_009494805.1 | Contig | 3.70095 | 44.9 | 55 | 4 | 2 | 77 | VZBG01 | USA: New York | 121 | 2946 | 3084 | Homo sapiens |
| 98 | iAK2716 | SAMN12583284 | GCA_009495085.1 | Contig | 3.68513 | 44.9 | 54 | 4 | 2 | 80 | VZAT01 | USA: New York | 119 | 2923 | 3063 | Homo sapiens |
| 99 | iAK2717 | SAMN12583291 | GCA_009494445.1 | Contig | 3.64203 | 44.9 | 56 | 4 | 2 | 90 | VZBW01 | USA: New York | 131 | 2900 | 3052 | Homo sapiens |
| 100 | iAK2718 | SAMN12583311 | GCA_009494175.1 | Contig | 3.6531 | 44.9 | 59 | 4 | 2 | 83 | VZCM01 | USA: New York | 132 | 2917 | 3065 | Homo sapiens |
| 101 | iAK278 | SAMN12583312 | GCA_009494135.1 | Contig | 3.42935 | 44.9 | 51 | 9 | 2 | 266 | VZCN01 | USA: New York | 419 | 2765 | 3093 | Homo sapiens |
| 102 | iAK279 | SAMN12583288 | GCA_009494565.1 | Contig | 3.68626 | 44.9 | 58 | 4 | 2 | 85 | VZBT01 | USA: New York | 107 | 2918 | 3067 | Homo sapiens |
| 103 | iAP1319 | SAMN12583313 | GCA_009494145.1 | Contig | 3.83658 | 44.6 | 45 | 6 | 2 | 71 | VZCO01 | USA: New York | 87 | 3090 | 3214 | Homo sapiens |
| 104 | iAP139 | SAMN12583254 | GCA_009495195.1 | Contig | 3.66419 | 44.9 | 48 | 7 | 2 | 65 | VZAM01 | USA: New York | 96 | 2932 | 3054 | Homo sapiens |
| 105 | iAP1411 | SAMN12583314 | GCA_009494105.1 | Contig | 3.68486 | 44.9 | 42 | 6 | 2 | 59 | VZCP01 | USA: New York | 74 | 2949 | 3058 | Homo sapiens |
| 106 | iAP1412 | SAMN12583273 | GCA_009494835.1 | Contig | 3.57563 | 44.9 | 50 | 7 | 2 | 59 | VZBE01 | USA: New York | 89 | 2834 | 2952 | Homo sapiens |
| 107 | iAP142 | SAMN12583315 | GCA_009494095.1 | Contig | 3.68001 | 44.9 | 43 | 7 | 2 | 56 | VZCQ01 | USA: New York | 83 | 2953 | 3061 | Homo sapiens |
| 108 | iAP144 | SAMN12583279 | GCA_009494735.1 | Contig | 3.81976 | 44.6 | 49 | 6 | 2 | 72 | VZBK01 | USA: New York | 103 | 3085 | 3214 | Homo sapiens |
| 109 | iAP145 | SAMN12583271 | GCA_009494895.1 | Contig | 3.8164 | 44.6 | 49 | 7 | 2 | 69 | VZBC01 | USA: New York | 93 | 3070 | 3197 | Homo sapiens |
| 110 | iAP146 | SAMN12583316 | GCA_009494035.1 | Contig | 3.58498 | 44.9 | 44 | 7 | 2 | 63 | VZCR01 | USA: New York | 95 | 2840 | 2956 | Homo sapiens |
| 111 | iAP171 | SAMN12583272 | GCA_009494875.1 | Contig | 3.66014 | 44.9 | 56 | 6 | 2 | 61 | VZBD01 | USA: New York | 90 | 2933 | 3058 | Homo sapiens |
| 112 | iAP188 | SAMN12583274 | GCA_009494845.1 | Contig | 3.66195 | 44.9 | 54 | 8 | 2 | 64 | VZBF01 | USA: New York | 93 | 2931 | 3059 | Homo sapiens |
| 113 | iAQ1144 | SAMN12583317 | GCA_009494055.1 | Contig | 3.80859 | 44.6 | 49 | 10 | 2 | 85 | VZCS01 | USA: New York | 174 | 3109 | 3255 | Homo sapiens |
| 114 | iAQ1149 | SAMN12583318 | GCA_009494045.1 | Contig | 3.73297 | 44.7 | 52 | 8 | 2 | 85 | VZCT01 | USA: New York | 151 | 3017 | 3164 | Homo sapiens |
| 115 | iAQ1172 | SAMN12583319 | GCA_009494015.1 | Contig | 3.96183 | 44.6 | 65 | 5 | 2 | 68 | VZCU01 | USA: New York | 125 | 3284 | 3424 | Homo sapiens |
| 116 | iAQ1173 | SAMN12583246 | GCA_009495355.1 | Contig | 3.1388 | 45.8 | 44 | 4 | 2 | 52 | VZAD01 | USA: New York | 100 | 2465 | 2567 | Homo sapiens |
| 117 | iAQ1174 | SAMN12583320 | GCA_009493995.1 | Contig | 3.94775 | 44.6 | 65 | 6 | 2 | 94 | VZCV01 | USA: New York | 180 | 3267 | 3434 | Homo sapiens |
| 118 | iAQ1179 | SAMN12583321 | GCA_009493895.1 | Contig | 3.91196 | 45.6 | 52 | 9 | 2 | 187 | VZCW01 | USA: New York | 415 | 3201 | 3451 | Homo sapiens |
| 119 | iAQ11815 | SAMN12583276 | GCA_009494765.1 | Contig | 3.803 | 44.6 | 54 | 11 | 2 | 80 | VZBH01 | USA: New York | 123 | 3079 | 3226 | Homo sapiens |
| 120 | iAQ1183 | SAMN12583322 | GCA_009493935.1 | Contig | 3.80873 | 44.6 | 53 | 10 | 2 | 75 | VZCX01 | USA: New York | 122 | 3086 | 3226 | Homo sapiens |
| 121 | iAU3127 | SAMN12583257 | GCA_009495155.1 | Contig | 3.75991 | 45.1 | 48 | 7 | 2 | 73 | VZAP01 | USA: New York | 199 | 2991 | 3120 | Homo sapiens |
| 122 | iK21316 | SAMN12583261 | GCA_009495055.1 | Contig | 3.5832 | 45.1 | 62 | 5 | 2 | 107 | VZAU01 | USA: New York | 129 | 2962 | 3137 | Homo sapiens |
| 123 | iK21513 | SAMN12583323 | GCA_009493945.1 | Contig | 3.53193 | 45.1 | 59 | 6 | 2 | 80 | VZCY01 | USA: New York | 123 | 2921 | 3067 | Homo sapiens |
| 124 | iK2152 | SAMN12583324 | GCA_009493955.1 | Contig | 3.56781 | 45 | 61 | 4 | 2 | 90 | VZCZ01 | USA: New York | 110 | 2950 | 3106 | Homo sapiens |
| 125 | iK21614 | SAMN12583325 | GCA_009493905.1 | Contig | 3.61773 | 45.1 | 60 | 6 | 2 | 79 | VZDA01 | USA: New York | 119 | 3012 | 3158 | Homo sapiens |
| 126 | iK21616 | SAMN12583262 | GCA_009495035.1 | Contig | 3.53305 | 45.2 | 71 | 6 | 2 | 83 | VZAV01 | USA: New York | 118 | 2877 | 3038 | Homo sapiens |
| 127 | Indica | SAMN07313576 | GCA_002224675.1 | Contig | 3.92056 | 45.4 | 70 | 23 | 2 | 51 | NMPZ01 | india | 88 | 3089 | 3265 | Homo sapiens |
| 128 | iP105 | SAMN12583260 | GCA_009495075.1 | Contig | 4.25632 | 44.7 | 57 | 7 | 2 | 115 | VZAS01 | USA: New York | 209 | 3478 | 3659 | Homo sapiens |
| 129 | iP54 | SAMN12583285 | GCA_009494595.1 | Contig | 4.07041 | 44.9 | 55 | 6 | 2 | 99 | VZBQ01 | USA: New York | 166 | 3297 | 3459 | Homo sapiens |
| 130 | iT211 | SAMN12583326 | GCA_009493835.1 | Contig | 3.39748 | 44.9 | 47 | 3 | 2 | 65 | VZDB01 | USA: New York | 86 | 2692 | 2809 | Homo sapiens |
| 131 | iT2110 | SAMN12583327 | GCA_009493815.1 | Contig | 3.40078 | 44.9 | 45 | 3 | 2 | 69 | VZDC01 | USA: New York | 91 | 2695 | 2814 | Homo sapiens |
| 132 | iT2112 | SAMN12583328 | GCA_009493825.1 | Contig | 3.40495 | 44.9 | 51 | 3 | 2 | 60 | VZDD01 | USA: New York | 90 | 2701 | 2817 | Homo sapiens |
| 133 | iT2113 | SAMN12583252 | GCA_009495285.1 | Contig | 3.39418 | 44.9 | 54 | 3 | 2 | 69 | VZAI01 | USA: New York | 91 | 2682 | 2810 | Homo sapiens |
| 134 | iT2117 | SAMN12583266 | GCA_009495235.1 | Contig | 3.38675 | 44.9 | 58 | 3 | 2 | 87 | VZAL01 | USA: New York | 126 | 2683 | 2833 | Homo sapiens |
| 135 | iT2119 | SAMN12583247 | GCA_009495385.1 | Contig | 3.39117 | 44.9 | 46 | 3 | 2 | 76 | VZAE01 | USA: New York | 91 | 2680 | 2807 | Homo sapiens |
| 136 | iT212 | SAMN12583329 | GCA_009493795.1 | Contig | 3.39685 | 44.9 | 46 | 3 | 2 | 61 | VZDE01 | USA: New York | 85 | 2690 | 2802 | Homo sapiens |
| 137 | iT2120 | SAMN12583281 | GCA_009494665.1 | Contig | 3.38923 | 44.9 | 50 | 3 | 2 | 59 | VZBN01 | USA: New York | 91 | 2682 | 2796 | Homo sapiens |
| 138 | iT213 | SAMN12583265 | GCA_009495095.1 | Contig | 3.39195 | 44.9 | 45 | 3 | 2 | 70 | VZAR01 | USA: New York | 107 | 2682 | 2802 | Homo sapiens |
| 139 | iT214 | SAMN12583249 | GCA_009495315.1 | Contig | 3.39848 | 44.9 | 50 | 3 | 2 | 72 | VZAG01 | USA: New York | 92 | 2691 | 2818 | Homo sapiens |
| 140 | iT218 | SAMN12583286 | GCA_009494605.1 | Contig | 3.38935 | 44.9 | 49 | 3 | 2 | 69 | VZBR01 | USA: New York | 93 | 2670 | 2793 | Homo sapiens |
| 141 | iT2213 | SAMN12583330 | GCA_009493805.1 | Contig | 3.40231 | 44.9 | 44 | 3 | 2 | 69 | VZDF01 | USA: New York | 86 | 2696 | 2814 | Homo sapiens |
| 142 | iT2510 | SAMN12583264 | GCA_009494985.1 | Contig | 3.38803 | 44.9 | 47 | 3 | 2 | 62 | VZAX01 | USA: New York | 102 | 2688 | 2802 | Homo sapiens |
| 143 | iT2513 | SAMN12583331 | GCA_009493745.1 | Contig | 3.41004 | 44.9 | 48 | 3 | 2 | 60 | VZDG01 | USA: New York | 87 | 2712 | 2825 | Homo sapiens |
| 144 | iT2515 | SAMN12583332 | GCA_009493705.1 | Contig | 3.40301 | 44.9 | 47 | 3 | 2 | 67 | VZDH01 | USA: New York | 84 | 2694 | 2813 | Homo sapiens |
| 145 | iT255 | SAMN12583248 | GCA_009495365.1 | Contig | 3.39599 | 44.9 | 49 | 3 | 2 | 70 | VZAF01 | USA: New York | 91 | 2683 | 2807 | Homo sapiens |
| 146 | iT257 | SAMN12583296 | GCA_009495005.1 | Contig | 3.38771 | 44.9 | 48 | 3 | 2 | 71 | VZAW01 | USA: New York | 98 | 2672 | 2796 | Homo sapiens |
| 147 | LKV-178-WT-2C | SAMN12619160 | GCA_009695805.1 | Contig | 3.51334 | 44.7 | 58 | 11 | 2 | 75 | VUNF01 | Germany: Freising | 77 | 2837 | 2983 | Sus scrofa |
| 148 | MCC688 | SAMN12147422 | GCA_018784085.1 | Scaffold | 3.48853 | 45.1 | 65 | 12 | 2 | 64 | WQNW01 | Ireland | 162 | 2791 | 2944 | Homo sapiens |
| 149 | MGYG-HGUT-03697 | SAMEA5853203 | GCA_902399905.1 | Scaffold | 3.9007 | 45.8 | 60 | 5 | 2 | 94 | CABOGV01 | China:Shenzhen | 138 | 3081 | 3242 |  |
| 150 | MSK.10.30 | SAMN19731845 | GCA_019131775.1 | Contig | 3.58101 | 45 | 62 | 4 | 2 | 63 | JAHOMZ01 | USA: New York | 131 | 2864 | 2995 | Homo sapiens |
| 151 | MSK.21.19 | SAMN19732062 | GCA_019127615.1 | Contig | 3.7042 | 44.9 | 59 | 8 | 2 | 74 | JAHOFD01 | USA: New York | 188 | 2909 | 3052 | Homo sapiens |
| 152 | MSK.21.22 | SAMN19732063 | GCA_019127725.1 | Contig | 3.75646 | 44.9 | 60 | 10 | 2 | 73 | JAHOFC01 | USA: New York | 193 | 2967 | 3112 | Homo sapiens |
| 153 | MSK.21.26 | SAMN19732066 | GCA_019127545.1 | Contig | 3.75347 | 44.9 | 60 | 9 | 2 | 75 | JAHOFB01 | USA: New York | 199 | 2967 | 3113 | Homo sapiens |
| 154 | MSK.21.28 | SAMN19732067 | GCA_019127455.1 | Contig | 3.80687 | 45 | 68 | 12 | 2 | 63 | JAHOFA01 | USA: New York | 174 | 2996 | 3141 | Homo sapiens |
| 155 | MSK.21.37 | SAMN19732072 | GCA_019127285.1 | Contig | 3.75727 | 44.9 | 57 | 11 | 2 | 78 | JAHOEV01 | USA: New York | 193 | 2968 | 3116 | Homo sapiens |
| 156 | MSK.21.44 | SAMN19732073 | GCA_019127575.1 | Contig | 4.20506 | 46 | 69 | 15 | 2 | 102 | JAHOEU01 | USA: New York | 389 | 3378 | 3566 | Homo sapiens |
| 157 | MSK.21.56 | SAMN19732076 | GCA_019127485.1 | Contig | 3.50127 | 45.6 | 47 | 3 | 2 | 84 | JAHOER01 | USA: New York | 265 | 2838 | 2974 | Homo sapiens |
| 158 | MSK.21.57 | SAMN19732077 | GCA_019127435.1 | Contig | 3.75695 | 44.9 | 60 | 9 | 2 | 79 | JAHOEQ01 | USA: New York | 200 | 2968 | 3118 | Homo sapiens |
| 159 | MSK.21.60 | SAMN19732078 | GCA_019127475.1 | Contig | 3.75908 | 44.9 | 60 | 11 | 2 | 79 | JAHOEP01 | USA: New York | 190 | 2970 | 3122 | Homo sapiens |
| 160 | MSK.21.61 | SAMN19732079 | GCA_019052415.1 | Contig | 3.76269 | 44.9 | 59 | 11 | 2 | 78 | JAHQUU01 | USA: New York | 178 | 2966 | 3116 | Homo sapiens |
| 161 | MSK.21.64 | SAMN19732081 | GCA_019127365.1 | Contig | 4.05805 | 44.7 | 66 | 15 | 3 | 96 | JAHOEO01 | USA: New York | 260 | 3270 | 3450 | Homo sapiens |
| 162 | MSK.21.65 | SAMN19732082 | GCA_019127395.1 | Contig | 4.11518 | 44.9 | 56 | 9 | 2 | 83 | JAHOEN01 | USA: New York | 238 | 3251 | 3401 | Homo sapiens |
| 163 | MSK.21.66 | SAMN19732083 | GCA_019127335.1 | Contig | 4.11585 | 44.9 | 54 | 7 | 2 | 82 | JAHOEM01 | USA: New York | 242 | 3245 | 3390 | Homo sapiens |
| 164 | MSK.21.71 | SAMN19732085 | GCA_019127415.1 | Contig | 3.77254 | 44.8 | 59 | 11 | 2 | 80 | JAHOEK01 | USA: New York | 188 | 2984 | 3136 | Homo sapiens |
| 165 | MSK.21.74 | SAMN19732087 | GCA_019127275.1 | Contig | 4.09647 | 44.9 | 56 | 7 | 2 | 90 | JAHOEI01 | USA: New York | 251 | 3232 | 3387 | Homo sapiens |
| 166 | MSK.21.77 | SAMN19732089 | GCA_019052335.1 | Contig | 3.88012 | 44.9 | 59 | 7 | 2 | 81 | JAHQUS01 | USA: New York | 227 | 3029 | 3178 | Homo sapiens |
| 167 | MSK.21.95 | SAMN19732096 | GCA_019127135.1 | Contig | 3.8848 | 44.9 | 58 | 5 | 2 | 84 | JAHOEA01 | USA: New York | 221 | 3021 | 3170 | Homo sapiens |
| 168 | OF03-3 | SAMN09736697 | GCA_003463005.1 | Scaffold | 4.0824 | 44.7 | 60 | 15 | 2 | 511 | QSCI01 | China:Shenzhen | 331 | 2984 | 3572 | Homo sapiens |
| 169 | OM04-1 | SAMN09736775 | GCA_003439165.1 | Scaffold | 3.90398 | 44.6 | 58 | 19 | 2 | 647 | QSUX01 | China:Shenzhen | 295 | 2792 | 3518 | Homo sapiens |
| 170 | OM06-11 | SAMN09736814 | GCA_003438885.1 | Scaffold | 3.61247 | 45.1 | 56 | 4 | 2 | 90 | QSUC01 | China:Shenzhen | 116 | 2794 | 2946 | Homo sapiens |
| 171 | P2A-3 | SAMN10723581 | GCA_009739305.1 | Contig | 3.82523 | 45.3 | 59 | 15 | 3 | 359 | SCPK01 | USA:Stanford University | 1 | 2720 | 3156 | Homo sapiens |
| 172 | P2B-2 | SAMN10723583 | GCA_009739345.1 | Contig | 3.791 | 45.3 | 59 | 15 | 2 | 365 | SCPM01 | USA:Stanford University | 1 | 2673 | 3114 | Homo sapiens |
| 173 | RHA03 | SAMN19158401 | GCA_019249635.1 | Contig | 3.54855 | 44.6 | 58 | 19 | 2 | 72 | JAHRGJ01 | Germany:Braunschweig | 101 | 2960 | 3111 | Homo sapiens |
| 174 | RPA01 | SAMN19158402 | GCA_019249715.1 | Contig | 3.47973 | 44.9 | 60 | 11 | 2 | 72 | JAHRGK01 | Germany:Braunschweig | 112 | 2825 | 2970 | Homo sapiens |
| 175 | SG-1727 | SAMN12628461 | GCA_021347525.1 | Contig | 3.74192 | 45.8 | 81 | 46 | 2 | 57 | VTYY01 | USA: Brooking | 286 | 3019 | 3205 | Homo sapiens |
| 176 | SG-1969 | SAMN12628462 | GCA_021347545.1 | Contig | 4.0396 | 45 | 71 | 40 | 2 | 68 | VTYX01 | USA: Brooking | 288 | 3214 | 3394 | Homo sapiens |
| 177 | SG-805 | SAMN12628460 | GCA_021347565.1 | Contig | 3.92781 | 45.9 | 84 | 46 | 2 | 74 | VTYZ01 | USA: Brooking | 513 | 3283 | 3488 | Homo sapiens |
| 178 | TF06-40 | SAMN09736895 | GCA_003437315.1 | Scaffold | 3.69357 | 44.9 | 68 | 5 | 2 | 91 | QSSA01 | China:Shenzhen | 100 | 2891 | 3057 | Homo sapiens |
| 179 | Y7FG | SAMN12329126 | GCA_016803185.1 | Scaffold | 4.12753 | 46.1 | 74 | 20 | 2 | 59 | WBJP01 | China:Xiamen | 155 | 3260 | 3415 | Homo sapiens |
| 180 | Y7XP | SAMN12329125 | GCA_016803255.1 | Scaffold | 4.12212 | 46 | 74 | 20 | 2 | 80 | WBJO01 | China:Xiamen | 181 | 3194 | 3370 | Homo sapiens |
| 181 | Y8CS | SAMN12329122 | GCA_016803435.1 | Contig | 3.67167 | 45.2 | 57 | 14 | 2 | 80 | WBJL01 | China:Xiamen | 81 | 2870 | 3023 | Homo sapiens |
| 182 | YF2 | SAMN12321477 | GCA_015074785.1 | Chromosome | 3.86403 | 44.9597 | 59 | 18 | 2 | 61 | WBJM01 | China:Xiamen | 2 | 3053 | 3193 | Homo sapiens |
| 183 | YG5 | SAMN12329124 | GCA_016803265.1 | Contig | 3.78678 | 44.8 | 56 | 18 | 2 | 69 | WBJN01 | China:Xiamen | 113 | 3033 | 3178 | Homo sapiens |

**Table S3 List of differential fecal metabolites between the diabetes group and other groups based on LC-MS.**

| Group | Metabolite | P value | FC | VIP | Trend |
| --- | --- | --- | --- | --- | --- |
| Metformin vs Diabetes | N-METHYL (-)EPHEDRINE | 0.000004 | 0.191552 | 1.305983 | down |
|  | Glutaconic acid | 0.000006 | 0.105017 | 1.834905 | down |
|  | cis-Aconitic acid | 0.000026 | 0.026074 | 1.651815 | down |
|  | 2-Furoic acid | 0.000037 | 0.023307 | 2.003621 | down |
|  | Acetaminophen glucuronide | 0.000051 | 0.164234 | 1.571829 | down |
|  | 6-bromo-3-(2-phenylethanhydrazonoyl)-2H-chromen-2-one | 0.000144 | 0.078304 | 1.580429 | down |
|  | 4-Butylresorcinol | 0.000149 | 0.185854 | 1.928154 | down |
|  | Sulfoacetic acid | 0.000168 | 0.252197 | 1.724410 | down |
|  | 2-Hydroxyphenylacetic acid | 0.000199 | 0.245336 | 1.667882 | down |
|  | 4-benzyl-N-(3,5-dichlorophenyl)-1,4-diazepane-1-carboxamide | 0.000248 | 0.135390 | 1.136133 | down |
|  | 5-Sulfosalicylic acid | 0.000326 | 0.098792 | 1.812700 | down |
|  | Homogentisic Acid | 0.000328 | 0.456235 | 1.574126 | down |
|  | 1,5,8-Trihydroxy-9-oxo-9H-xanthen-3-yl beta-D-glucopyranoside | 0.000341 | 0.086478 | 1.473120 | down |
|  | 3-(3-nitrophenyl)-2-phenylacrylic acid | 0.000612 | 0.062188 | 1.858455 | down |
|  | Quercetin | 0.000737 | 0.295307 | 1.281364 | down |
|  | Stachyose | 0.000758 | 0.212878 | 1.641870 | down |
|  | Adipic acid | 0.000930 | 0.270505 | 1.531390 | down |
|  | δ-Gluconic acid δ-lactone | 0.001022 | 0.542490 | 1.116049 | down |
|  | (3-Methoxy-4-hydroxyphenyl)ethylene glycol sulfate | 0.001343 | 0.125093 | 1.338639 | down |
|  | D-Raffinose | 0.001365 | 0.264744 | 1.250437 | down |
|  | 3-[(2-thienylthio)methyl]benzoic acid | 0.001420 | 0.324982 | 1.070205 | down |
|  | N-Formylkynurenine | 0.001519 | 0.290584 | 1.587233 | down |
|  | 2-(Formylamino)Benzoic Acid | 0.001583 | 0.185846 | 1.666908 | down |
|  | L-Threonic acid | 0.001624 | 0.089774 | 1.716697 | down |
|  | Purine | 0.001898 | 0.394458 | 1.378660 | down |
|  | L-Ascorbate | 0.001921 | 0.164582 | 1.367947 | down |
|  | allantoate | 0.002279 | 0.093504 | 1.688833 | down |
|  | 3-Indoxyl sulphate | 0.002291 | 0.038459 | 1.548433 | down |
|  | 7-Hydroxy-4-chromone | 0.002360 | 0.251653 | 1.502947 | down |
|  | Citric acid | 0.002383 | 0.015472 | 2.007917 | down |
|  | 4-Hydroxy-3- methoxyphenylglycol sulfate | 0.002427 | 0.070425 | 1.727337 | down |
|  | 3-Methyladipic acid | 0.002618 | 0.411847 | 1.391880 | down |
|  | D-(+)-Malic acid | 0.002875 | 0.242408 | 1.806357 | down |
|  | Orotidine | 0.002967 | 0.217310 | 1.912515 | down |
|  | Gluconic acid | 0.003062 | 0.477325 | 1.385670 | down |
|  | Pimelic acid | 0.003088 | 0.405011 | 1.357859 | down |
|  | 2-Ketoadipic acid | 0.003118 | 0.144256 | 1.003041 | down |
|  | 2-Oxobutyric acid | 0.003150 | 0.292170 | 1.863669 | down |
|  | Phenylacetylglycine | 0.003251 | 0.161671 | 1.398138 | down  （附表2）  （附表2）  （附表2） |
|  | 1-Methyluric acid | 0.003706 | 0.341532 | 1.085457 | down |
|  | Hexanoyl glycine | 0.003879 | 0.200053 | 1.145345 | down |
|  | Allantoic acid | 0.003945 | 0.120894 | 1.582304 | down |
|  | L-Cysteinesulfinic acid | 0.004223 | 0.323458 | 1.769904 | down |
|  | gamma-Nonanolactone | 0.004264 | 0.338508 | 1.471725 | down |
|  | D-α-Hydroxyglutaric acid | 0.004501 | 0.250922 | 1.655246 | down |
|  | Homocysteic acid | 0.004529 | 0.494103 | 1.057872 | down |
|  | 5,6-dihydroxyindole | 0.004623 | 0.256769 | 1.489358 | down |
|  | Pseudouridine | 0.004790 | 0.310800 | 1.691493 | down |
|  | Indole-2-carboxylic acid | 0.004798 | 0.406309 | 1.299302 | down |
|  | DL-Malic acid | 0.004858 | 0.237944 | 1.727048 | down |
|  | L-Ascorbic acid 2-sulfate | 0.005038 | 0.104403 | 1.678167 | down |
|  | Ureidosuccinic acid | 0.005071 | 0.125548 | 1.891633 | down |
|  | Catechol | 0.005096 | 0.076555 | 1.673132 | down |
|  | N-Acetyl-α-D-glucosamine 1-phosphate | 0.006138 | 0.182225 | 1.064765 | down |
|  | N-Tigloylglycine | 0.006534 | 0.227062 | 1.201274 | down |
|  | Allantoin  Metformin  vs Diabetes | 0.006791 | 0.114469 | 1.699215 | down |
|  | 7-Methylxanthine | 0.006986 | 0.125324 | 1.731701 | down |
|  | N-Acetyl-L-glutamine | 0.006989 | 0.351356 | 1.687476 | down |
|  | Capryloylglycine | 0.007300 | 0.167749 | 1.273126 | down |
|  | H-Trp-NH2.HCl | 0.008488 | 0.239547 | 1.587824 | down |
|  | N'5-[3-(trifluoromethyl)benzoyl]-2,1,3-benzoxadiazole-5-carbohydrazide | 0.009240 | 0.406938 | 1.250010 | down |
|  | D-Xylonic Acid Lithium Salt | 0.009418 | 0.233705 | 1.681163 | down |
|  | 6-methylimidazo[2,1-b][1,3]thiazole-5-carbohydrazide | 0.010918 | 0.514645 | 1.310326 | down |
|  | Adipamide | 0.010986 | 0.348749 | 1.714756 | down |
|  | Fumaric acid | 0.011998 | 0.148105 | 1.806462 | down |
|  | 3-(2-Hydroxyethyl)indole | 0.012341 | 0.312745 | 1.190922 | down |
|  | D-Threose | 0.012414 | 0.300366 | 1.261698 | down |
|  | 2-(1H-1,2,3-benzotriazol-1-yl)-N-(2,3-dihydro-1H-inden-2-yl)acetamide | 0.012685 | 0.401253 | 1.112532 | down |
|  | 4-Hydroxybenzylcyanide | 0.013367 | 0.131894 | 1.789902 | down |
|  | Hippuric acid | 0.013445 | 0.123265 | 1.792610 | down |
|  | Levodopa | 0.013907 | 0.129478 | 1.790587 | down |
|  | Hydroxyglutaric acid | 0.013947 | 0.604707 | 1.298185 | down |
|  | N-Acetyl-L-phenylalanine | 0.014566 | 0.407359 | 1.116055 | down |
|  | alpha-Benzylsuccinic acid | 0.015896 | 0.322606 | 1.655305 | down |
|  | 5-(3-chloro-4-methylanilino)-1-methyl-1H-pyrazol-3-ol | 0.017726 | 0.301127 | 1.117505 | down |
|  | N-Acetyl-L-histidine | 0.018342 | 0.570926 | 1.191252 | down |
|  | 15-Epiprostaglandin E1 | 0.019346 | 0.189028 | 1.455666 | down |
|  | D-(-)-Fructose | 0.019744 | 0.131505 | 1.459797 | down |
|  | 4-Hydroxy-6-methyl-2-pyrone | 0.020134 | 0.456912 | 1.294188 | down |
|  | Cinnamoylglycine | 0.021615 | 0.256894 | 1.083992 | down |
|  | Epinephrine | 0.022429 | 0.302055 | 1.121562 | down |
|  | Υ-Aminobutyric acid (GABA) | 0.026743 | 0.330311 | 1.392981 | down |
|  | Monobutyl phthalate | 0.026900 | 0.507252 | 1.291417 | down |
|  | N6-Succinyl Adenosine | 0.027578 | 0.298581 | 1.346946 | down |
|  | L-Glutamic acid | 0.029461 | 0.351767 | 1.433215 | down |
|  | D-(+)-Mannose | 0.030037 | 0.222933 | 1.132039 | down |
|  | 4-Hydroxybenzaldehyde | 0.034951 | 0.440238 | 1.116860 | down |
|  | Erythronolactone | 0.039123 | 0.307853 | 1.384761 | down |
|  | N-α-Acetyl-L-asparagine | 0.047284 | 0.486010 | 1.042846 | down |
|  | OxPG (16:0-20:3+1O(1Cyc)) | 0.047347 | 0.269165 | 1.324058 | down |
|  | Salvinorin B | 0.000020 | 3.314276 | 1.808900 | up |
|  | (±)9(10)-DiHOME | 0.000159 | 2.077797 | 1.551650 | up |
|  | 8-iso-15-keto Prostaglandin F2α | 0.000163 | 2.816395 | 1.759018 | up |
|  | 3-[2-(1,3,5-trimethyl-1H-pyrazol-4-yl)hydrazono]pentane-2,4-dione | 0.000465 | 2.051425 | 1.727201 | up |
|  | α-Aspartylphenylalanine | 0.000542 | 3.509206 | 1.320732 | up |
|  | Suberic acid | 0.000558 | 2.621001 | 1.654728 | up |
|  | dopaquinone  Metformin  vs Diabetes | 0.000753 | 3.863371 | 1.997378 | up |
|  | 5-Methyl-dl-tryptophan | 0.000838 | 3.230202 | 1.941486 | up |
|  | 6-Keto-prostaglandin f1alpha | 0.000992 | 1.847646 | 1.384778 | up |
|  | Lysopc 14:0 | 0.001201 | 1.906654 | 1.087220 | up |
|  | Tetracycline | 0.001320 | 2.343136 | 1.398016 | up |
|  | 3,4,5-trihydroxycyclohex-1-ene-1-carboxylic acid | 0.001433 | 2.261609 | 1.805536 | up |
|  | (±)10(11)-EpDPA | 0.001444 | 2.708496 | 1.564935 | up |
|  | Ip7G | 0.001469 | 5.137178 | 1.738683 | up |
|  | Kynurenic acid | 0.001502 | 2.164469 | 1.551266 | up |
|  | Pyridoxine O-Glucoside | 0.001610 | 3.436376 | 1.519530 | up |
|  | Jasmonic acid | 0.001664 | 1.750626 | 1.443669 | up |
|  | Glycoursodeoxycholic acid | 0.002094 | 2.960985 | 1.621604 | up |
|  | 13,14-Dihydro-15-keto Prostaglandin E1 | 0.002212 | 1.771534 | 1.429939 | up |
|  | 3,8,9-trihydroxy-10-propyl-3,4,5,8,9,10-hexahydro-2H-oxecin-2-one | 0.002334 | 2.627972 | 1.678633 | up |
|  | (±)12(13)-DiHOME | 0.002549 | 3.357492 | 1.901269 | up |
|  | 1-methyl-N-(4-piperidinophenyl)-1H-imidazole-4-sulfonamide | 0.002793 | 1.935352 | 1.420448 | up |
|  | (±)7(8)-DiHDPA | 0.002908 | 2.254522 | 1.377847 | up |
|  | Phe-Phe | 0.003068 | 11.38571 | 2.040849 | up |
|  | Spiculisporic Acid | 0.003209 | 1.721742 | 1.256476 | up |
|  | indole-5,6-quinone | 0.003337 | 2.741302 | 1.214578 | up |
|  | Asp-glu | 0.004025 | 2.734082 | 1.211012 | up |
|  | 2-Hydroxy-2-methyl-3-buten-1-yl beta-D-glucopyranoside | 0.005628 | 3.844590 | 1.545114 | up |
|  | N-lactoyl-phenylalanine | 0.006409 | 2.698116 | 1.552497 | up |
|  | 11-keto Testosterone (CRM) | 0.006575 | 1.861995 | 1.287480 | up |
|  | Prostaglandin K1 | 0.006837 | 1.694998 | 1.092351 | up |
|  | Prostaglandin B2 | 0.008315 | 2.803024 | 1.609856 | up |
|  | 3-Hydroxybutyric acid | 0.008692 | 9.846805 | 1.831456 | up |
|  | 17α-Hydroxyprogesterone | 0.008753 | 2.689149 | 1.232273 | up |
|  | 7-Ketolithocholic acid | 0.008800 | 2.882460 | 1.422104 | up |
|  | LPC 14:0 | 0.011936 | 5.613041 | 1.412099 | up |
|  | Leucine-enkephalin | 0.013975 | 1.793073 | 1.497736 | up |
|  | Esculin | 0.015553 | 3.856304 | 1.362072 | up |
|  | geranyl pp | 0.017221 | 2.134688 | 1.035957 | up |
|  | Mesalamine | 0.018313 | 2.500281 | 1.398947 | up |
|  | Citrulline | 0.021902 | 2.274479 | 1.034309 | up |
|  | D-2-Aminoadipic acid | 0.021933 | 2.804296 | 1.198793 | up |
|  | Phenylpyruvic acid | 0.023181 | 3.217036 | 1.537716 | up |
|  | (±)11-HETE | 0.023234 | 1.835635 | 1.066384 | up |
|  | 1-benzyl-3-(2-methylphenyl)-3,7-dihydro-1H-purine-2,6-dione | 0.023634 | 1.962581 | 1.089070 | up |
|  | Chenodeoxycholic acid-3-beta-D-glucuronide | 0.025419 | 3.166310 | 1.681424 | up |
|  | Tauroursodeoxycholic acid Dihydrate | 0.025804 | 1.854682 | 1.146878 | up |
|  | Hexadecanedioic acid | 0.025978 | 3.466411 | 1.494516 | up |
|  | Gly-Tyr-Ala | 0.027031 | 2.005892 | 1.086656 | up |
|  | α,α-Trehalose | 0.027556 | 3.318163 | 1.548337 | up |
|  | β-D-Glucopyranuronic acid | 0.027750 | 2.405478 | 1.350672 | up |
|  | Prostaglandin A1 ethyl ester | 0.028382 | 1.797217 | 1.032321 | up |
|  | 9-HpOTrE  Metformin  vs Diabetes | 0.028772 | 2.470741 | 1.208410 | up |
|  | Dodecanedioic acid | 0.029034 | 1.646850 | 1.012517 | up |
|  | P-Coumaroyl Agmatine | 0.029078 | 3.543833 | 1.577533 | up |
|  | 4-Methylphenol | 0.030729 | 2.364849 | 1.442797 | up |
|  | 10-Hydroxydecanoic acid | 0.031453 | 2.299128 | 1.321330 | up |
|  | Monensin | 0.034153 | 1.898976 | 1.160006 | up |
|  | D-Gluconic acid | 0.038154 | 4.331670 | 1.240752 | up |
|  | Ferulic acid | 0.042392 | 2.102171 | 1.133954 | up |
|  | D-Ala-D-Ala | 0.043619 | 2.850821 | 1.185399 | up |
|  | 2,3-Dinor-TXB2 | 0.045171 | 1.788825 | 1.254296 | up |
|  | UDP-N-acetylglucosamine | 0.046839 | 1.998769 | 1.335063 | up |
|  | 2,5-Furandicarboxylic acid | 0.000003 | 0.033301 | 1.816382 | down |
|  | D-threo-Isocitric acid | 0.000009 | 0.014730 | 1.915152 | down |
|  | P-Aminohippuric Acid | 0.000010 | 0.105506 | 1.530134 | down |
|  | 7,8-Dihydrobiopterin | 0.000027 | 0.125499 | 1.481738 | down |
|  | 2-(acetylamino)-3-[4-(acetylamino)phenyl]acrylic acid | 0.000034 | 0.171203 | 1.688029 | down |
|  | 2,6-Xylidine | 0.000038 | 0.141833 | 1.350493 | down |
|  | 3-Methoxytyramine | 0.000043 | 0.214235 | 1.346250 | down |
|  | DL-Stachydrine | 0.000047 | 0.097527 | 1.768176 | down |
|  | GPH | 0.000056 | 0.058313 | 1.963978 | down |
|  | alpha-Ketoglutaric acid | 0.000062 | 0.021063 | 1.891129 | down |
|  | L(-)-Carnitine | 0.000080 | 0.129782 | 1.753264 | down |
|  | Argininosuccinic acid | 0.000149 | 0.231671 | 1.748700 | down |
|  | Citraconic acid | 0.000186 | 0.163546 | 1.664133 | down |
|  | Sinapinic acid | 0.000215 | 0.114630 | 1.757243 | down |
|  | 1-Methylguanine | 0.000245 | 0.203493 | 1.132192 | down |
|  | Trigonelline | 0.000248 | 0.107308 | 2.005050 | down |
|  | Ethyl sorbate | 0.000262 | 0.430191 | 1.386597 | down |
|  | 4-acetyl-4-(ethoxycarbonyl)heptanedioic acid | 0.000273 | 0.048915 | 1.795539 | down |
|  | Gluconolactone | 0.000274 | 0.201571 | 1.440354 | down |
|  | 3-Indoleacetonitrile | 0.000275 | 0.233829 | 1.704414 | down |
|  | Imidazolelactic acid | 0.000300 | 0.208912 | 1.888620 | down |
|  | N-α-L-Acetyl-arginine | 0.000311 | 0.279024 | 1.779373 | down |
|  | 4-Hydroxymandelonitrile | 0.000561 | 0.235530 | 1.144530 | down |
|  | 4-[1-(acetyloxy)prop-2-en-1-yl]-2-methoxyphenyl 2-methylpropanoate | 0.000588 | 0.070237 | 1.523960 | down |
|  | HET0016 | 0.000596 | 0.176742 | 1.797088 | down |
|  | N-Acetylserotonin | 0.000700 | 0.391476 | 1.170123 | down |
|  | Quinoline  Metformin  vs Diabetes | 0.000735 | 0.162313 | 1.330210 | down |
|  | 1-Methylnicotinamide | 0.000831 | 0.060917 | 1.766623 | down |
|  | 6 β-Hydroxycortisol | 0.000840 | 0.226318 | 1.407563 | down |
|  | DL-Arginine | 0.000875 | 0.128554 | 1.553444 | down |
|  | 2'-O-Methyluridine | 0.000935 | 0.110765 | 1.801490 | down |
|  | Melezitose | 0.000961 | 0.168699 | 1.425750 | down |
|  | Kynurenic acid O-hexside | 0.000990 | 0.281538 | 1.209093 | down |
|  | Cryptotanshinone | 0.001036 | 0.367513 | 1.426101 | down |
|  | 4-(allyloxy)-1,2-dihydroquinolin-2-one | 0.001085 | 0.400494 | 1.292191 | down |
|  | 1,7-bis(4-hydroxyphenyl)heptan-3-one | 0.001261 | 0.090576 | 1.797769 | down |
|  | 4-Guanidinobutyric acid | 0.001338 | 0.054832 | 1.963444 | down |
|  | Radicicol | 0.001417 | 0.077808 | 1.738200 | down |
|  | α-Methylhistamine | 0.001425 | 0.210709 | 1.000114 | down |
|  | DL-Carnitine | 0.001502 | 0.131905 | 1.850617 | down |
|  | Acipimox | 0.001613 | 0.483248 | 1.099617 | down |
|  | 4-oxo-4-[(1-phenylethyl)amino]but-2-enoic acid | 0.001816 | 0.210315 | 1.532389 | down |
|  | 5-Methoxytryptophol | 0.001868 | 0.251961 | 1.454219 | down |
|  | 4,7-diphenyl[1,10]phenanthroline | 0.001928 | 0.264555 | 1.357907 | down |
|  | 2,3,4-Trihydroxybenzoic acid | 0.002150 | 0.296038 | 1.106304 | down |
|  | D-(-)-Glutamine | 0.002251 | 0.304514 | 1.239529 | down |
|  | 3,5-Dihydroxyphenylglycine | 0.002371 | 0.380697 | 1.158464 | down |
|  | 4-oxo-5-phenylpentanoic acid | 0.002497 | 0.347166 | 1.417861 | down |
|  | DL-2-(acetylamino)-3-phenylpropanoic acid | 0.002510 | 0.525912 | 1.165701 | down |
|  | N-Acetyl-D-lactosamine | 0.002735 | 0.460485 | 1.254562 | down |
|  | Delta-Tridecalactone | 0.003055 | 0.196050 | 1.420604 | down |
|  | N1-(2-amino-2-oxoethyl)-2-(isopropylthio)acetamide | 0.003240 | 0.216992 | 1.203198 | down |
|  | N-Acetylanthranilic acid | 0.003274 | 0.150897 | 1.612144 | down |
|  | 4-(1H-pyrazol-1-yl)-N,N-bis(2-pyridinylmethyl)benzenesulfonamide | 0.003419 | 0.313110 | 1.439363 | down |
|  | L-Aspartic acid | 0.003479 | 0.158115 | 1.623281 | down |
|  | Ureidoisobutyric Acid | 0.003711 | 0.176523 | 1.223055 | down |
|  | Carnosine | 0.003782 | 0.295108 | 1.558645 | down |
|  | L-Homocitrulline | 0.004013 | 0.305696 | 1.107567 | down |
|  | Creatine | 0.004057 | 0.292356 | 1.375129 | down |
|  | 4-Guanidinobutanoic acid | 0.004152 | 0.065229 | 1.924463 | down |
|  | N-Carbamyl-L-glutamicacid | 0.005182 | 0.292322 | 1.637852 | down |
|  | 2-Methoxyresorcinol | 0.005635 | 0.269881 | 1.589134 | down |
|  | Phenylglyoxylic acid | 0.005827 | 0.177421 | 1.296591 | down |
|  | Υ-L-Glutamyl-L-glutamic acid | 0.006048 | 0.416870 | 1.367154 | down |
|  | N1-[4-hydroxy-6-(methoxymethyl)pyrimidin-2-yl]acetamide  Metformin  vs Diabetes | 0.006346 | 0.263997 | 1.667531 | down |
|  | N6,N6,N6-Trimethyl-L-lysine | 0.006362 | 0.258084 | 1.551247 | down |
|  | lithocholic acid 3-sulfate sodium salt | 0.006516 | 0.332286 | 1.569270 | down |
|  | Methyl 3-indolyacetate | 0.006584 | 0.401818 | 1.262453 | down |
|  | 5-hydroxy-4-methoxy-5,6-dihydro-2H-pyran-2-one | 0.006684 | 0.294301 | 1.281698 | down |
|  | Lysine Butyrate | 0.007101 | 0.252042 | 1.605974 | down |
|  | D-Erythrose 4-phosphate | 0.007316 | 0.212734 | 1.553685 | down |
|  | Physostigmine | 0.007464 | 0.247757 | 1.115105 | down |
|  | L-Asparagine | 0.008087 | 0.141728 | 1.324938 | down |
|  | Isophorone | 0.008630 | 0.295111 | 1.419989 | down |
|  | N-Methylhydantoin | 0.009119 | 0.197960 | 1.234273 | down |
|  | Creatinine | 0.009183 | 0.125298 | 1.785176 | down |
|  | Cortisone | 0.009988 | 0.196833 | 1.685049 | down |
|  | 3-Hydroxyanthranilic Acid | 0.010869 | 0.411084 | 1.264713 | down |
|  | Maltotetraose | 0.011704 | 0.262069 | 1.161377 | down |
|  | PC (14:0e/17:0) | 0.012058 | 0.202479 | 1.186535 | down |
|  | Estropipate | 0.012229 | 0.351130 | 1.318443 | down |
|  | 2-morpholino-1-phenyl-1-ethanol | 0.012899 | 0.225051 | 1.263526 | down |
|  | Hypotaurine | 0.014350 | 0.196421 | 2.005420 | down |
|  | Nicotinuric acid | 0.015106 | 0.188887 | 1.374654 | down |
|  | 5-Methylcytosine | 0.016135 | 0.435859 | 1.697624 | down |
|  | Tropolone | 0.016213 | 0.099851 | 1.716020 | down |
|  | cis-7-Hexadecenoic Acid | 0.016327 | 0.479690 | 1.791794 | down |
|  | N-Acetyl-L-leucine | 0.016755 | 0.236340 | 1.186418 | down |
|  | 1-(3,4-dimethoxyphenyl)ethan-1-one oxime | 0.016783 | 0.529625 | 1.360082 | down |
|  | Inositol | 0.017302 | 0.193240 | 1.062223 | down |
|  | Fumonisin B1 | 0.018556 | 0.342408 | 1.030819 | down |
|  | D-Glucosamine | 0.019935 | 0.165161 | 1.580592 | down |
|  | Cortisol | 0.020357 | 0.147633 | 1.472468 | down |
|  | L-Histidine | 0.020972 | 0.337575 | 1.469189 | down |
|  | Uracil 1-beta-D-arabinofuranoside | 0.022896 | 0.293061 | 1.440058 | down |
|  | β-Nicotinamide mononucleotide | 0.023691 | 0.281572 | 1.081847 | down |
|  | D-(+)-Glucose | 0.024859 | 0.154995 | 1.189547 | down |
|  | SDMA | 0.026139 | 0.186871 | 1.868139 | down |
|  | Phosphocholine | 0.027127 | 0.106840 | 1.217795 | down |
|  | Cytidine-5'-monophosphate | 0.027324 | 0.345703 | 1.273075 | down |
|  | α-Lactose | 0.028527 | 0.182614 | 1.062895 | down |
|  | 1-Methylhistidine | 0.029018 | 0.246284 | 1.479779 | down |
|  | YNK | 0.030037 | 0.098121 | 1.260514 | down |
|  | S-Adenosylmethionine | 0.030277 | 0.204479 | 1.744983 | down |
|  | Nicotinate ribonucleoside | 0.033207 | 0.301160 | 1.141015 | down |
|  | Indirubin | 0.033958 | 0.043431 | 1.250384 | down |
|  | 4-[1-(dimethylamino)ethylidene]-2-phenyl-1,3-oxazol-5(4H)-one | 0.035875 | 0.468594 | 1.044072 | down |
|  | QMK  Metformin  vs Diabetes | 0.035955 | 0.236722 | 1.149571 | down |
|  | L-Leucyl-L-alanine Hydrate | 0.038458 | 0.536581 | 1.485093 | down |
|  | Trehalose | 0.041721 | 0.296391 | 1.004022 | down |
|  | L-Pyroglutamic acid | 0.041748 | 0.359334 | 1.121375 | down |
|  | 3-Methyladenine | 0.044197 | 0.458420 | 1.227197 | down |
|  | Bilirubin | 0.047323 | 0.132983 | 1.382132 | down |
|  | Ergosterol peroxide | 0.000102 | 2.250467 | 1.622112 | up |
|  | Adrenosterone | 0.000103 | 2.164471 | 1.683088 | up |
|  | Tetramethylpyrazine | 0.000126 | 49.44214 | 1.946614 | up |
|  | T-2 Triol | 0.000324 | 8.807172 | 2.054496 | up |
|  | 4-oxododecanedioic acid | 0.000421 | 2.497045 | 1.606071 | up |
|  | 3-Acetyl-2,5-dimethylfuran | 0.000438 | 2.028755 | 1.849922 | up |
|  | 15-Keto prostaglandin F1α | 0.000630 | 2.017111 | 1.900052 | up |
|  | 15-OxoEDE | 0.000729 | 2.100057 | 1.857104 | up |
|  | 3-hydroxy-2-octylpentanedioic acid | 0.000777 | 2.059440 | 1.705388 | up |
|  | 11-Deoxy prostaglandin F1α | 0.001018 | 2.491412 | 1.512444 | up |
|  | Diosgenin | 0.001022 | 1.801173 | 1.432478 | up |
|  | Thromoboxane B1 | 0.001124 | 1.878095 | 1.554521 | up |
|  | methyl 2-[(2-acetyl-3-oxo-1-butenyl)amino]acetate | 0.001140 | 2.271017 | 1.850652 | up |
|  | O-7460 | 0.001200 | 2.519964 | 1.477883 | up |
|  | 2-Thio-acetyl MAGE | 0.001327 | 5.131779 | 1.139636 | up |
|  | Andrographolide | 0.001336 | 1.860192 | 1.709780 | up |
|  | (9cis)-Retinal | 0.001338 | 3.170767 | 1.656915 | up |
|  | 3,5-Diiodo-L-thyronine | 0.001409 | 5.774057 | 1.448177 | up |
|  | 1-(3-ethyl-2,4-dihydroxy-6-methoxyphenyl)butan-1-one | 0.001426 | 1.858074 | 1.423260 | up |
|  | Betulin | 0.001499 | 2.117760 | 1.395405 | up |
|  | PC (7:0/8:0) | 0.001512 | 3.101826 | 2.095006 | up |
|  | Estradiol | 0.001760 | 1.797786 | 1.753364 | up |
|  | Mirtazapine-d3 | 0.002145 | 2.344711 | 1.887652 | up |
|  | 4-Pyridoxic acid | 0.002149 | 2.430406 | 1.850428 | up |
|  | 11-Deoxy prostaglandin F1β | 0.002228 | 2.272517 | 1.610788 | up |
|  | Adenine | 0.002236 | 3.019581 | 1.135126 | up |
|  | ethyl 5-methoxy-2-methyl-1-phenyl-1H-indole-3-carboxylate | 0.002368 | 3.062696 | 1.786178 | up |
|  | PC (14:1e/2:0) | 0.002402 | 4.715535 | 1.825011 | up |
|  | (+/-)11(12)-DiHET | 0.002615 | 1.701233 | 1.122544 | up |
|  | (3R)-8-hydroxy-3-(4-methoxyphenyl)-3,4-dihydro-1H-2-benzopyran-1-one | 0.002665 | 3.433844 | 1.800328 | up |
|  | 2-(3,4-dihydroxyphenyl)acetamide | 0.002722 | 2.495573 | 1.897035 | up |
|  | L-lysine | 0.003188 | 2.289270 | 1.727059 | up |
|  | PDMP | 0.003240 | 3.799377 | 2.000186 | up |
|  | 3-[4-methyl-1-(2-methylpropanoyl)-3-oxocyclohexyl]butanoic acid | 0.003262 | 2.312886 | 1.852676 | up |
|  | 2,6-Di-tert-butyl-1,4-benzoquinone | 0.003314 | 2.299061 | 1.841397 | up |
|  | Pregnenolone | 0.003332 | 3.242004 | 1.613725 | up |
|  | Homo-Gamma-Linolenic Acid (C20:3)  Metformin  vs Diabetes | 0.003391 | 2.080387 | 1.642970 | up |
|  | 20-Hydroxy prostaglandin F2α | 0.003601 | 3.421854 | 1.960646 | up |
|  | (12Z)-9,10,11-trihydroxyoctadec-12-enoic acid | 0.003624 | 2.172864 | 1.919837 | up |
|  | WKK | 0.003630 | 2.270073 | 1.608849 | up |
|  | 5-(hydroxymethyl)-4-methoxy-2,5-dihydrofuran-2-one | 0.003760 | 2.264893 | 1.461672 | up |
|  | LysoPE 18:2 | 0.003796 | 3.239645 | 1.642427 | up |
|  | 5beta-Androstane-3,17-dione | 0.003826 | 1.987817 | 1.996875 | up |
|  | Anacardic acid | 0.004442 | 4.743684 | 1.888244 | up |
|  | Tryptophanol | 0.004553 | 2.413036 | 1.652494 | up |
|  | Methionine | 0.004579 | 6.414711 | 1.659454 | up |
|  | (11E,15Z)-9,10,13-trihydroxyoctadeca-11,15-dienoic acid | 0.004963 | 1.619422 | 1.452031 | up |
|  | Glycohyocholic acid Sodium salt | 0.005016 | 3.067735 | 1.866509 | up |
|  | α-Lapachone | 0.005463 | 3.424689 | 1.882055 | up |
|  | Styrene | 0.005474 | 2.247683 | 1.622350 | up |
|  | Choline | 0.006144 | 2.129618 | 1.132465 | up |
|  | 3-(3,4-Dihydroxyphenyl)-2-Methylalanine | 0.006499 | 2.269248 | 1.192196 | up |
|  | 4-oxo-4-(5,6,7,8-tetrahydronaphthalen-1-ylamino)but-2-enoic acid | 0.006976 | 2.078204 | 1.583537 | up |
|  | 17beta-Trenbolone | 0.007011 | 2.254841 | 1.731210 | up |
|  | 5,6-dimethoxy-2-(2-methoxyphenyl)-4H-chromen-4-one | 0.008048 | 3.485439 | 1.649856 | up |
|  | p-Mentha-1,3,8-triene | 0.008966 | 3.395024 | 1.954728 | up |
|  | 8-Hydroxyquinoline | 0.011410 | 1.806489 | 1.556932 | up |
|  | Traumatic acid | 0.011764 | 4.201907 | 1.530190 | up |
|  | Prostaglandin E2-1-glyceryl ester | 0.011800 | 2.051013 | 1.201299 | up |
|  | Dehydroepiandrosterone (DHEA) | 0.012527 | 2.391494 | 1.075151 | up |
|  | Lysopa 16:0 | 0.012564 | 2.435140 | 1.247019 | up |
|  | Lagochilin | 0.012985 | 2.188642 | 1.623616 | up |
|  | Hydroquinone | 0.013730 | 1.705203 | 1.428398 | up |
|  | Deoxycorticosterone | 0.014544 | 2.984192 | 1.466528 | up |
|  | 1,4-dihydroxy-1,4-dimethyl-7-(propan-2-ylidene)-decahydroazulen-6-one | 0.014578 | 1.873430 | 1.740979 | up |
|  | DL-α-Aminocaprylic acid | 0.014754 | 2.349561 | 1.602165 | up |
|  | Coenzyme Q2 | 0.014853 | 2.203269 | 1.517400 | up |
|  | 6-Methoxy-2-naphthoic acid | 0.015144 | 3.581732 | 1.765648 | up |
|  | 2-Methoxyestrone | 0.015196 | 2.352994 | 1.465440 | up |
|  | Valerophenone  Metformin  vs Diabetes | 0.015209 | 2.898951 | 1.344078 | up |
|  | Glycolithocholic acid | 0.015288 | 3.311771 | 1.307283 | up |
|  | octadec-9-ynoic acid | 0.015746 | 3.764795 | 1.431715 | up |
|  | ACar 18:2 | 0.015925 | 2.505553 | 1.459824 | up |
|  | (+/-)11(12)-EET | 0.015932 | 1.518110 | 1.182963 | up |
|  | ACar 20:4 | 0.015983 | 3.266318 | 1.193978 | up |
|  | Taurochenodeoxycholic Acid (sodium salt) | 0.016958 | 3.047624 | 2.020795 | up |
|  | Sedanolide | 0.018242 | 1.600080 | 1.084803 | up |
|  | (5E)-7-methylidene-10-oxo-4-(propan-2-yl)undec-5-enoic acid | 0.018270 | 2.785028 | 1.459617 | up |
|  | XLR11 N-(4-hydroxypentyl) metabolite-d5 | 0.018506 | 1.777480 | 1.110199 | up |
|  | 2,2-dimethyl-6,7-di[(4-nitrobenzyl)oxy]chroman-4-one | 0.019444 | 4.157051 | 1.348040 | up |
|  | N-Acetylornithine | 0.020870 | 5.566964 | 1.452611 | up |
|  | 15-Deoxy-Δ12,14-prostaglandin J2-2-glycerol ester | 0.021137 | 2.452747 | 1.023414 | up |
|  | Fasciculic acid B | 0.021487 | 2.126667 | 1.497042 | up |
|  | Dihydrokawain | 0.023745 | 1.541147 | 1.023506 | up |
|  | Nicotinic acid | 0.024484 | 2.450398 | 1.621985 | up |
|  | 8-Isoprostaglandin F1α | 0.024825 | 1.886502 | 1.128087 | up |
|  | Linolelaidic Acid (C18:2N6T) | 0.025365 | 2.241099 | 1.233359 | up |
|  | (S)-AL 8810 | 0.025468 | 1.819175 | 1.260337 | up |
|  | Cafestol | 0.026141 | 2.424418 | 1.167781 | up |
|  | (2R)-2-[(2R,5S)-5-[(2S)-2-hydroxybutyl]oxolan-2-yl]propanoic acid | 0.027017 | 1.778418 | 1.467271 | up |
|  | DKK | 0.027595 | 2.616615 | 1.011919 | up |
|  | Tetranor-12(S)-HETE | 0.030180 | 1.851041 | 1.162574 | up |
|  | Carvone | 0.030669 | 1.618050 | 1.339540 | up |
|  | Boc-beta-cyano-L-alanine | 0.031535 | 2.932867 | 1.023065 | up |
|  | Methyl dihydrojasmonate | 0.031583 | 1.513321 | 1.234647 | up |
|  | 9-Oxo-10(E),12(E)-octadecadienoic acid | 0.031741 | 1.802268 | 1.333847 | up |
|  | ACar 16:1 | 0.032509 | 1.905791 | 1.194600 | up |
|  | Cholecalciferol | 0.032638 | 2.203513 | 1.100026 | up |
|  | 1,3-Dihydro-1,3,3-trimethyl-2H-indol-2-ylidene acetaldehyde | 0.032778 | 2.042914 | 1.436523 | up |
|  | Isoferulic acid | 0.036075 | 2.063414 | 1.016555 | up |
|  | 13,14-dihydro-15-keto Prostaglandin A2 | 0.037415 | 1.604163 | 1.205007 | up |
|  | 6-Gingerol  Metformin  vs Diabetes | 0.037813 | 1.872873 | 1.252111 | up |
|  | 4-Methoxybenzaldehyde | 0.037899 | 1.690491 | 1.266314 | up |
|  | Androsterone | 0.039941 | 1.696227 | 1.438868 | up |
|  | 1-(7-methoxy-2-oxo-2H-chromen-8-yl)-3-methyl-2-oxobutyl acetate | 0.040019 | 2.496802 | 1.244907 | up |
|  | Thymine | 0.040666 | 2.281072 | 1.110753 | up |
|  | Mezlocillin | 0.040871 | 4.595681 | 1.676343 | up |
|  | 4-(2,3-dihydro-1,4-benzodioxin-6-yl)butanoic acid | 0.041785 | 1.518946 | 1.197788 | up |
|  | Ginsenoside Rg2 | 0.044482 | 3.476359 | 1.327608 | up |
|  | Prostaglandin A3 | 0.045995 | 1.427373 | 1.166763 | up |
|  | Uracil | 0.046217 | 3.121612 | 1.703259 | up |
|  | Melanin | 0.048004 | 1.693878 | 1.123399 | up |
|  | 16-Heptadecyne-1,2,4-triol | 0.048037 | 3.046622 | 1.452909 | up |
|  | 4-(3,4-dimethoxyphenyl)-3-methyl-1H-pyrazol-5-amine | 0.049860 | 1.786550 | 1.461528 | up |
| HF2123 vs. Diabetes | S-Adenosyl-L-methionine | 0.003100 | 0.598162 | 2.770368 | down |
|  | DL-Homoserine | 0.023921 | 0.577852 | 1.675871 | down |
|  | L-(+)-Citrulline | 0.034327 | 0.595791 | 1.495168 | down |
|  | Oleanolic acid | 0.036207 | 0.518685 | 2.310241 | down |
|  | Biliverdin | 0.038727 | 0.422598 | 1.842870 | down |
|  | 1-hydroxy-1-(4-methoxyphenyl)propan-2-yl 4-methoxybenzoate | 0.039140 | 0.780117 | 1.214456 | down |
|  | Guanine | 0.004534 | 3.843614 | 2.943970 | up |
|  | Tetramethylpyrazine | 0.005781 | 16.93554 | 2.862223 | up |
|  | Ethyl chrysanthemumate | 0.010148 | 1.570979 | 2.668628 | up |
|  | Methyl-2-aminobenzoate | 0.016186 | 1.538092 | 1.750060 | up |
|  | gamma-Glutamyltyrosine | 0.018474 | 1.520120 | 2.383853 | up |
|  | SKK | 0.018827 | 1.915557 | 1.602093 | up |
|  | trans-4-Phenyl-4-piperidinocyclohexanol | 0.020107 | 2.303087 | 1.315648 | up |
|  | 3-(5-phenyl-1,3-oxazol-2-yl)-4-(trifluoromethyl)pyridine | 0.023889 | 2.922799 | 2.324872 | up |
|  | α-Linolenoyl ethanolamide | 0.024697 | 1.334033 | 1.941793 | up |
|  | Fumonisin B1 | 0.026003 | 3.157325 | 1.980355 | up |
|  | MJN110 | 0.032280 | 2.087275 | 2.471691 | up |
|  | Octadeca-11E,13E,15Z-trienoic acid | 0.033787 | 1.457494 | 1.684163 | up |
|  | Deisopropylatrazine | 0.036442 | 4.287449 | 2.776203 | up |
|  | methyl 3,4,5-trihydroxycyclohex-1-ene-1-carboxylate | 0.040273 | 1.394797 | 1.531049 | up |
|  | Mezlocillin | 0.042861 | 4.470829 | 3.563749 | up |
|  | Biotin | 0.046655 | 1.665939 | 1.734373 | up |
|  | D-2-Aminoadipic acid | 0.004441 | 0.443683 | 2.293925 | down |
|  | Rutin | 0.034619 | 0.398108 | 1.549054 | down |
|  | N-Oleoyl dopamine | 0.035320 | 0.277932 | 2.816605 | down |
|  | N-Acetylglycine | 0.044211 | 0.692645 | 1.686243 | down |
|  | D-Xylonic Acid Lithium Salt  HF2123  vs Diabetes | 0.004535 | 1.605740 | 1.154163 | up |
|  | Glu-Gln | 0.009937 | 1.713944 | 1.638960 | up |
|  | 4-Methylphenol | 0.014944 | 1.377315 | 1.296301 | up |
|  | FAHFA (22:5/22:5) | 0.026228 | 2.399147 | 2.037889 | up |
|  | L-Ascorbate | 0.029061 | 3.049606 | 1.929094 | up |
|  | N4-Acetylcytidine | 0.030449 | 2.000063 | 2.373268 | up |
|  | 4-[2-(2-oxo-1-imidazolidinyl)ethyl]-1lambda~6~,4-thiazinane-1,1-dione | 0.033842 | 1.555634 | 1.503970 | up |
|  | Feruloyl Putrescine | 0.034298 | 2.550088 | 1.865773 | up |
|  | Sulfoacetic acid | 0.035322 | 1.487734 | 1.167653 | up |
|  | D-Glucarate | 0.040590 | 1.674052 | 1.908782 | up |
|  | Guanosine | 0.041416 | 3.063687 | 2.708493 | up |
|  | (2R)-2,3-Dihydroxypropanoic acid | 0.047488 | 1.752585 | 1.503255 | up |
| HF1478 vs. Diabetes | Daidzein | 0.001890 | 0.395999 | 2.322824 | down |
|  | R-1 Methanandamide phosphate | 0.003717 | 0.317789 | 1.757885 | down |
|  | Nicotinamide mononucleotide | 0.006723 | 0.193934 | 2.302012 | down |
|  | 2-morpholino-1-phenyl-1-ethanol | 0.008473 | 0.238706 | 2.127812 | down |
|  | 5-[(E)-2-(3,5-dihydroxyphenyl)ethenyl]-2-methoxybenzene-1,3-diol | 0.009184 | 0.506194 | 1.847879 | down |
|  | RPK | 0.009831 | 0.391000 | 1.095135 | down |
|  | Kynurenic acid O-hexside | 0.010497 | 0.415356 | 1.398126 | down |
|  | 4',7-Dihydroxyflavanone | 0.013456 | 0.504487 | 1.979167 | down |
|  | XLR11 N-(4-hydroxypentyl) metabolite | 0.013895 | 0.422285 | 1.394192 | down |
|  | Artemisinin | 0.014533 | 0.527693 | 2.294291 | down |
|  | 6 -Hydroxycortisol | 0.018278 | 0.315910 | 2.037600 | down |
|  | 5-[(10Z)-14-(3,5-dihydroxyphenyl)tetradec-10-en-1-yl]benzene-1,3-diol | 0.018947 | 0.208094 | 1.928584 | down |
|  | HET0016 | 0.020516 | 0.422696 | 1.565902 | down |
|  | Bilirubin | 0.022434 | 0.123885 | 1.930101 | down |
|  | Cortisone | 0.033313 | 0.319419 | 2.080599 | down |
|  | 2-[(3S)-1-(3,4-Difluorobenzyl)-3-pyrrolidinyl]-1,3-benzoxazole | 0.035714 | 0.525201 | 1.420330 | down |
|  | DL-Panthenol | 0.036709 | 0.746230 | 1.134378 | down |
|  | 2-{[(4,5-dimethoxy-2-nitrophenethyl)imino]methyl}phenol | 0.037267 | 0.342438 | 2.039276 | down |
|  | Phylloquinone | 0.042344 | 0.420914 | 2.967991 | down |
|  | 4-acetyl-4-(ethoxycarbonyl)heptanedioic acid | 0.042377 | 0.342768 | 1.235305 | down |
|  | Uracil | 0.005405 | 2.003752 | 1.956700 | up |
|  | FMH | 0.006678 | 3.223238 | 2.429083 | up |
|  | Homo-Gamma-Linolenic Acid (C20:3) | 0.008800 | 1.594171 | 1.805535 | up |
|  | DKK | 0.009165 | 2.880294 | 1.993090 | up |
|  | trans-4-Phenyl-4-piperidinocyclohexanol | 0.009655 | 4.881725 | 1.922355 | up |
|  | 3-Nitro-L-Tyrosine | 0.010585 | 2.033793 | 1.175686 | up |
|  | Tetramethylpyrazine | 0.012132 | 41.22314 | 2.775528 | up |
|  | SKK | 0.012769 | 2.370656 | 1.647231 | up |
|  | acetoacetate | 0.013976 | 1.794726 | 2.038818 | up |
|  | N-Acetylhistamine | 0.014217 | 1.872498 | 1.727209 | up |
|  | Cytidine | 0.019611 | 2.835913 | 2.751206 | up |
|  | 2'-Deoxyguanosine | 0.019637 | 3.192076 | 2.200288 | up |
|  | Asp-Phe methyl ester | 0.019762 | 2.331999 | 2.197523 | up |
|  | Lagochilin | 0.020070 | 1.467874 | 1.388125 | up |
|  | LNH | 0.021229 | 1.907962 | 1.793778 | up |
|  | MJN110 | 0.021327 | 2.393566 | 2.378558 | up |
|  | 3-(dimethylamino)-1-[4-(phenylsulfonyl)phenyl]prop-2-en-1-one  HF1478  vs Diabetes | 0.021593 | 1.477861 | 1.638791 | up |
|  | Guanine | 0.023987 | 2.844237 | 1.781225 | up |
|  | L-Alanyl-L-proline | 0.025459 | 2.732037 | 1.812579 | up |
|  | 3-pentadecyl-4,5,6,7-tetrahydrobenzo[d]isoxazol-4-one oxime | 0.034123 | 1.457252 | 1.337240 | up |
|  | Prostaglandin E2-1-glyceryl ester | 0.034684 | 1.753480 | 1.601331 | up |
|  | Valylproline | 0.036125 | 2.616398 | 1.558659 | up |
|  | Deoxyinosine | 0.041857 | 3.003767 | 2.260681 | up |
|  | LPH | 0.042472 | 3.842344 | 2.126608 | up |
|  | RNH | 0.043005 | 1.885649 | 2.050770 | up |
|  | UR-144 N-(2-hydroxypentyl) metabolite | 0.043255 | 3.104119 | 1.706034 | up |
|  | 3-(3,4-Dihydroxyphenyl)-2-Methylalanine | 0.043353 | 2.345536 | 2.006738 | up |
|  | Mezlocillin | 0.043906 | 4.391662 | 2.831483 | up |
|  | 4-Pyridoxic acid | 0.046127 | 1.397832 | 1.210578 | up |
|  | 2-Benzyl-5-[(3S)-1-isopropyl-3-pyrrolidinyl]-1,3,4-oxadiazole | 0.047361 | 2.734784 | 1.218607 | up |
|  | PNK | 0.049655 | 2.347793 | 1.504123 | up |
|  | 3-[(2-thienylthio)methyl]benzoic acid | 0.004393 | 0.397845 | 1.405749 | down |
|  | Homocysteic acid | 0.004942 | 0.625584 | 1.091257 | down |
|  | Corticosterone | 0.005634 | 0.271037 | 1.172187 | down |
|  | Phenylacetylglycine | 0.010066 | 0.200921 | 2.028268 | down |
|  | 3-Indoxyl sulphate | 0.010847 | 0.095123 | 1.838294 | down |
|  | Orotic acid | 0.012136 | 0.661373 | 1.448791 | down |
|  | Catechol | 0.014653 | 0.286004 | 1.292847 | down |
|  | (3-Methoxy-4-hydroxyphenyl)ethylene glycol sulfate | 0.017048 | 0.313305 | 1.231613 | down |
|  | Methylgallate | 0.017302 | 0.412309 | 2.641989 | down |
|  | Monobutyl phthalate | 0.017678 | 0.637620 | 1.272959 | down |
|  | 4-Hydroxy-3- methoxyphenylglycol sulfate | 0.018132 | 0.257431 | 1.460281 | down |
|  | 3-(3-nitrophenyl)-2-phenylacrylic acid | 0.018616 | 0.249017 | 1.556755 | down |
|  | OxPG (18:0-22:6+4O) | 0.021530 | 0.390152 | 2.174994 | down |
|  | Fumaric acid | 0.026302 | 0.389649 | 1.284879 | down |
|  | Rutin | 0.027516 | 0.264614 | 1.873696 | down |
|  | 5-Sulfosalicylic acid | 0.037177 | 0.455910 | 1.032783 | down |
|  | 4-benzyl-N-(3,5-dichlorophenyl)-1,4-diazepane-1-carboxamide | 0.000561 | 5.368733 | 1.574978 | up |
|  | 2'-Deoxyinosine | 0.005036 | 3.826112 | 2.662876 | up |
|  | 3-Hydroxy-3-methylglutaric acid | 0.005057 | 1.388461 | 1.431899 | up |
|  | Glu-Gln | 0.005899 | 1.778279 | 1.255275 | up |
|  | UDP-N-acetylglucosamine | 0.006508 | 1.882147 | 2.052289 | up |
|  | Feruloyl Putrescine | 0.012525 | 3.964673 | 1.962031 | up |
|  | PG (3:0/16:1)  HF1478  vs Diabetes | 0.013491 | 2.851614 | 2.616878 | up |
|  | 6-Hydroxymelatonin | 0.016905 | 2.997362 | 1.825740 | up |
|  | Phe-Pro | 0.019320 | 2.123567 | 1.789288 | up |
|  | Leucylproline | 0.020116 | 2.408797 | 1.397172 | up |
|  | Phe-Phe | 0.020522 | 2.913490 | 1.483498 | up |
|  | 4-Methoxycinnamic Acid | 0.022302 | 1.525797 | 1.573089 | up |
|  | FAHFA (2:0/16:0) | 0.022954 | 3.655550 | 2.237784 | up |
|  | 5-Methyl-dl-tryptophan | 0.023940 | 1.798846 | 1.525051 | up |
|  | Chenodeoxycholic acid-3-beta-D-glucuronide | 0.026811 | 1.588570 | 1.173659 | up |
|  | (±)11-HETE | 0.029995 | 1.652510 | 1.427311 | up |
|  | 3-Hydroxysebacic acid | 0.032963 | 1.314868 | 1.199683 | up |
|  | Stearic acid | 0.034164 | 2.188095 | 1.416635 | up |
|  | α,α-Trehalose | 0.036481 | 3.157894 | 2.244514 | up |
|  | N1-(3-amino-4-chlorophenyl)-2-[2,4-di(tert-pentyl)phenoxy]acetamide | 0.036801 | 2.518583 | 1.419143 | up |
|  | PC (16:0/18:1) | 0.039540 | 2.547855 | 1.635811 | up |
|  | Dimetghyl 4-Hydroxyisophthalate | 0.040375 | 1.361553 | 1.084931 | up |
|  | Geranylgeranyl pyrophosphate | 0.040668 | 3.925953 | 2.354860 | up |
|  | Jasmonic acid | 0.041672 | 1.311068 | 1.100557 | up |
|  | FAHFA (2:0/18:0) | 0.042033 | 4.148300 | 2.039114 | up |
|  | 3-[2-(1,3,5-trimethyl-1H-pyrazol-4-yl)hydrazono]pentane-2,4-dione | 0.044041 | 1.576124 | 1.644894 | up |
|  | 13,14-Dihydro prostaglandin E1 | 0.044473 | 1.469429 | 1.398101 | up |
|  | Arachidonic acid | 0.045151 | 1.369338 | 1.066585 | up |
|  | NSI-189 | 0.048657 | 3.125762 | 2.251437 | up |
| Hf2130 vs. Diabetes | Corticosterone | 0.002951 | 0.241589 | 1.335939 | down |
|  | O-Phospho-L-serine | 0.016756 | 0.242312 | 2.613442 | down |
|  | 4-Butylresorcinol | 0.024603 | 0.379648 | 2.002178 | down |
|  | Orotidine | 0.027950 | 0.388719 | 1.903236 | down |
|  | 8(R)-Hydroxy-(5Z,9E,11Z,14Z)-eicosatetraenoic acid | 0.032190 | 0.275551 | 2.585000 | down |
|  | D-Threose | 0.037063 | 0.364269 | 1.722778 | down |
|  | Adenosine diphosphate ribose | 0.037731 | 0.246738 | 2.393082 | down |
|  | O-Acetylserine | 0.041740 | 0.461116 | 2.640177 | down |
|  | Pantetheine | 0.043230 | 0.108684 | 1.784303 | down |
|  | 2-Oxobutyric acid | 0.045468 | 0.497740 | 1.753443 | down |
|  | 7-Hydroxy-4-chromone | 0.045754 | 0.481410 | 1.352765 | down |
|  | 23-Nordeoxycholic acid | 0.046105 | 0.581727 | 1.279514 | down |
|  | Uridine monophosphate (UMP) | 0.049280 | 0.179697 | 2.348010 | down |
|  | 2-[(3S)-1-(Cyclohexylmethyl)-3-pyrrolidinyl]-5-fluoro-1H-benzimidazole | 0.003804 | 2.560541 | 1.232418 | up |
|  | Gamma-Glu-Leu | 0.005327 | 2.947429 | 1.711762 | up |
|  | Leucylproline | 0.007816 | 2.386053 | 1.412289 | up |
|  | N1-(3-amino-4-chlorophenyl)-2-[2,4-di(tert-pentyl)phenoxy]acetamide | 0.014489 | 2.479826 | 1.406279 | up |
|  | Guanosine | 0.018769 | 2.812348 | 1.992855 | up |
|  | Tyrosylalanine | 0.024409 | 2.394102 | 1.665905 | up |
|  | 2'-Deoxyinosine | 0.025235 | 2.147170 | 1.494845 | up |
|  | Oleoyl-L-α-lysophosphatidic acid | 0.027703 | 6.168211 | 2.918045 | up |
|  | PG (2:0/16:0) | 0.028303 | 3.153807 | 2.488201 | up |
|  | PG (2:0/18:1) | 0.039341 | 4.563473 | 1.514670 | up |
|  | Ferulic acid | 0.040905 | 2.382759 | 1.969795 | up |
|  | N4-Acetylcytidine | 0.043029 | 1.870742 | 1.534285 | up |
|  | 2,6-Xylidine | 0.003499 | 0.187217 | 1.967051 | down |
|  | 5-Methylcytosine | 0.006244 | 0.485652 | 2.279187 | down |
|  | Adenine | 0.006938 | 0.507944 | 1.146283 | down |
|  | S-Adenosyl-L-methionine | 0.009784 | 0.652179 | 1.759987 | down |
|  | Cytidine-5'-monophosphate | 0.011050 | 0.412031 | 1.586914 | down |
|  | Sphingosine (d18:1) | 0.013915 | 0.630875 | 1.881529 | down |
|  | gamma-Glutamylleucine | 0.014067 | 0.415234 | 2.302166 | down |
|  | Adenosine | 0.014090 | 0.379786 | 2.166689 | down |
|  | 1-[4-hydroxy-3-(3-methylbut-2-en-1-yl)phenyl]ethan-1-one | 0.014209 | 0.681765 | 1.256359 | down |
|  | Oleanolic acid | 0.019978 | 0.580516 | 1.382157 | down |
|  | Artemisinin | 0.020766 | 0.713761 | 1.078214 | down |
|  | Cortisone | 0.022775 | 0.286810 | 2.137213 | down |
|  | D-(-)-Glutamine | 0.029903 | 0.515940 | 1.101074 | down |
|  | Bilirubin | 0.030909 | 0.197112 | 1.242615 | down |
|  | β-Nicotinamide mononucleotide | 0.031100 | 0.191653 | 2.472931 | down |
|  | Cortisol | 0.040263 | 0.243702 | 1.801184 | down |
|  | Maltol | 0.041581 | 0.427006 | 1.032022 | down |
|  | Hypotaurine | 0.043480 | 0.349301 | 2.086742 | down |
|  | 4-Acetamidobutyric Acid | 0.044386 | 0.376175 | 2.954600 | down |
|  | Ethyl sorbate | 0.045972 | 0.584132 | 1.560003 | down |
|  | Υ-L-Glutamyl-L-glutamic acid | 0.046118 | 0.658090 | 1.002498 | down |
|  | 3-(3,4-Dihydroxyphenyl)-2-Methylalanine | 0.000046 | 3.369372 | 2.926504 | up |
|  | UR-144 N-(2-hydroxypentyl) metabolite | 0.000595 | 3.191385 | 1.803702 | up |
|  | trans-4-Phenyl-4-piperidinocyclohexanol | 0.000695 | 4.211123 | 1.750897 | up |
|  | Coumarin | 0.001006 | 1.842805 | 1.087421 | up |
|  | IMK | 0.001151 | 2.178848 | 1.465970 | up |
|  | Asp-Phe methyl ester | 0.002348 | 2.609044 | 2.427155 | up |
|  | Thr-Leu  HF2130  vs Diabetes | 0.002485 | 3.749496 | 2.460458 | up |
|  | Ala-Val | 0.002850 | 2.834296 | 1.245892 | up |
|  | Serotonin | 0.003041 | 1.725336 | 1.394518 | up |
|  | FLK | 0.003713 | 2.941488 | 1.388011 | up |
|  | Valylproline | 0.004428 | 2.906980 | 1.731027 | up |
|  | Prostaglandin E2-1-glyceryl ester | 0.005729 | 2.365577 | 2.303664 | up |
|  | tert-Butyl N-[1-(aminocarbonyl)-3-methylbutyl]carbamate | 0.005949 | 3.400978 | 1.428947 | up |
|  | Glycocholic acid hydrate | 0.006222 | 3.703310 | 1.954918 | up |
|  | (2E)-6-hydroxy-2-methyl-6-(4-methylphenyl)hept-2-enoic acid | 0.006401 | 2.670744 | 2.215394 | up |
|  | 2-Hydroxycinnamic acid | 0.006626 | 1.662594 | 1.009297 | up |
|  | LNH | 0.007358 | 2.393870 | 2.249896 | up |
|  | L-(-)-Methionine | 0.007462 | 3.151667 | 2.264675 | up |
|  | VLK | 0.007574 | 2.713588 | 1.448001 | up |
|  | SKK | 0.008428 | 2.246426 | 1.525953 | up |
|  | PNK | 0.008748 | 2.102842 | 1.364065 | up |
|  | trans-3-Hexenoic acid | 0.008797 | 3.735399 | 2.774475 | up |
|  | VPH | 0.009174 | 5.345114 | 1.867256 | up |
|  | DLK | 0.009337 | 2.174085 | 1.641537 | up |
|  | Guanine | 0.010713 | 3.270782 | 1.974038 | up |
|  | PLH | 0.011597 | 2.301968 | 1.515288 | up |
|  | L-Phenylalanine | 0.012024 | 1.607747 | 1.199776 | up |
|  | FNK | 0.012060 | 2.573753 | 1.597295 | up |
|  | AKB48 N-(4-hydroxypentyl) metabolite | 0.013580 | 3.698771 | 2.020325 | up |
|  | Alanyltyrosine | 0.014285 | 2.202922 | 1.523299 | up |
|  | L-(-)-alpha-Amino-epsilon-Caprolactam | 0.014300 | 3.477482 | 2.233458 | up |
|  | Cytidine | 0.015653 | 2.699501 | 2.492402 | up |
|  | KNK | 0.015977 | 2.302383 | 1.537743 | up |
|  | 3-hydroxy-3-methylpentanedioic acid | 0.016822 | 2.005228 | 2.125400 | up |
|  | Epinephrine bitartrate | 0.017124 | 1.706097 | 1.877369 | up |
|  | Lysopc 18:2 | 0.017277 | 2.658840 | 1.645130 | up |
|  | 3-(dimethylamino)-1-[4-(phenylsulfonyl)phenyl]prop-2-en-1-one | 0.017314 | 1.698278 | 2.018112 | up |
|  | Pregnenolone | 0.018033 | 1.597748 | 1.080847 | up |
|  | Gly-Phe | 0.018407 | 2.822766 | 1.627866 | up |
|  | Diosgenin | 0.018439 | 1.493742 | 1.536155 | up |
|  | LPH | 0.018929 | 3.536548 | 2.009132 | up |
|  | 2-(2-amino-3-methylbutanamido)-3-phenylpropanoic acid | 0.019294 | 2.251769 | 1.267895 | up |
|  | 3'-Hydroxystanozolol | 0.020167 | 3.011463 | 1.426007 | up |
|  | INK | 0.020931 | 2.734665 | 2.058338 | up |
|  | VLH | 0.021479 | 2.868538 | 1.476422 | up |
|  | L-Alanyl-L-proline | 0.022850 | 2.423726 | 1.603367 | up |
|  | Glycyl-L-leucine | 0.022928 | 2.854762 | 1.481160 | up |
|  | 6-chloro-5-fluoro-1H-benzo[d]imidazole | 0.024197 | 1.630333 | 1.402360 | up |
|  | Val-Ser | 0.025379 | 2.424836 | 1.225542 | up |
|  | Ala-Ile | 0.025469 | 2.414088 | 1.169397 | up |
|  | ANK | 0.027096 | 1.965009 | 1.058789 | up |
|  | DPK | 0.028177 | 2.071434 | 1.093509 | up |
|  | Phenylethanolamine | 0.033416 | 2.017364 | 1.373719 | up |
|  | Gly-Val | 0.034576 | 2.727568 | 1.468717 | up |
|  | 2-[(3S)-1-Benzyl-3-pyrrolidinyl]-1,3-benzothiazole  HF2130  vs Diabetes | 0.034789 | 1.348085 | 1.465032 | up |
|  | 4-(3-Methyl-5-oxo-4,5-dihydro-1H-pyrazol-1-yl)benzoic acid | 0.035011 | 1.781181 | 1.924230 | up |
|  | ELK | 0.036442 | 2.131203 | 1.081817 | up |
|  | EMH | 0.036593 | 2.310409 | 1.434913 | up |
|  | Loxoprofen | 0.036944 | 2.844970 | 1.585733 | up |
|  | Tetramethylpyrazine | 0.039699 | 5.398125 | 1.222696 | up |
|  | IMH | 0.039984 | 2.896851 | 1.582475 | up |
|  | Asp-Phe | 0.040319 | 2.344389 | 1.979084 | up |
|  | 2'-Deoxyguanosine | 0.040718 | 3.265300 | 1.976296 | up |
|  | 17beta-Trenbolone | 0.041666 | 1.633455 | 1.660679 | up |
|  | 2,6-Dihydroxypurine | 0.041918 | 3.408883 | 2.089533 | up |
|  | TLK | 0.042470 | 3.098436 | 1.370894 | up |
|  | 7-Aminoflunitrazepam-d7 | 0.042582 | 1.749085 | 1.704137 | up |
|  | PDMP | 0.044491 | 1.818961 | 1.433765 | up |
|  | 2-Methoxyestrone | 0.044632 | 1.524682 | 1.182480 | up |
|  | (6E)-7-(2H-1,3-benzodioxol-5-yl)-1-(piperidin-1-yl)hept-6-en-1-one | 0.045267 | 2.357769 | 1.765385 | up |
|  | 3-(propan-2-yl)-octahydropyrrolo[1,2-a]pyrazine-1,4-dione | 0.045617 | 2.456105 | 1.278346 | up |
|  | Isoferulic acid | 0.046612 | 2.214064 | 1.738667 | up |
|  | Mezlocillin | 0.048661 | 3.876923 | 2.505487 | up |
|  | pentane-1,2,3,4,5-pentol | 0.048729 | 1.943777 | 1.847796 | up |
|  | 3-Hydroxypicolinic acid | 0.048858 | 1.405767 | 1.854416 | up |

**Table S4 Specific genes of strain HF2130 compared to the other three strains**

|  | type | length_bp | gene | EC_number | COG | product |
| --- | --- | --- | --- | --- | --- | --- |
| 1 | CDS | 1563 |  |  |  | hypothetical protein |
| 2 | CDS | 1605 | rhaR |  |  | HTH-type transcriptional activator RhaR |
| 3 | CDS | 366 |  | 3.2.1.23 | COG3250 | Beta-galactosidase BoGH2A |
| 4 | CDS | 1140 | araJ |  | COG2814 | Putative transporter AraJ |
| 5 | CDS | 564 |  |  |  | hypothetical protein |
| 6 | CDS | 2430 |  |  |  | hypothetical protein |
| 7 | CDS | 534 |  |  |  | hypothetical protein |
| 8 | CDS | 963 |  |  |  | hypothetical protein |
| 9 | CDS | 384 |  |  |  | hypothetical protein |
| 10 | CDS | 381 |  | 5.1.3.29 |  | L-fucose mutarotase |
| 11 | CDS | 2037 |  |  |  | hypothetical protein |
| 12 | CDS | 975 |  |  |  | hypothetical protein |
| 13 | CDS | 2667 | mgtB | 7.2.2.14 | COG0474 | Magnesium-transporting ATPase, P-type 1 |
| 14 | CDS | 1377 | arlS | 2.7.13.3 |  | Signal transduction histidine-protein kinase ArlS |
| 15 | CDS | 687 | cusR |  | COG0745 | Transcriptional regulatory protein CusR |
| 16 | CDS | 837 |  |  |  | hypothetical protein |
| 17 | CDS | 654 | sapB |  | COG1285 | Protein SapB |
| 18 | CDS | 732 |  |  |  | hypothetical protein |
| 19 | CDS | 774 | yknY | 3.6.3.- | COG1136 | putative ABC transporter ATP-binding protein YknY |
| 20 | CDS | 1182 | macA_1 |  | COG0845 | Macrolide export protein MacA |
| 21 | CDS | 1335 |  |  |  | hypothetical protein |
| 22 | CDS | 690 |  |  |  | hypothetical protein |
| 23 | CDS | 1962 | dnaG_1 | 2.7.7.- |  | DNA primase |
| 24 | CDS | 1425 | clsA | 2.7.8.- | COG1502 | Major cardiolipin synthase ClsA |
| 25 | CDS | 813 |  |  |  | hypothetical protein |
| 26 | CDS | 489 |  |  |  | hypothetical protein |
| 27 | CDS | 1539 | recD2_1 | 3.6.4.12 |  | ATP-dependent RecD-like DNA helicase |
| 28 | CDS | 1560 |  |  |  | hypothetical protein |
| 29 | tRNA | 83 |  |  |  | tRNA-Tyr(gta) |
| 30 | CDS | 1383 |  |  |  | hypothetical protein |
| 31 | CDS | 543 |  |  |  | hypothetical protein |
| 32 | CDS | 645 |  |  |  | hypothetical protein |
| 33 | CDS | 501 |  |  |  | hypothetical protein |
| 34 | CDS | 1644 |  |  |  | hypothetical protein |
| 35 | CDS | 348 |  |  |  | hypothetical protein |
| 36 | CDS | 912 | parB |  | COG1475 | putative chromosome-partitioning protein ParB |
| 37 | CDS | 765 | soj_1 | 3.6.-.- | COG1192 | Sporulation initiation inhibitor protein Soj |
| 38 | CDS | 744 | surE | 3.1.3.5 | COG0496 | 5'-nucleotidase SurE |
| 39 | tRNA | 72 |  |  |  | tRNA-Gln(ctg) |
| 40 | tRNA | 72 |  |  |  | tRNA-Gln(ctg) |
| 41 | CDS | 1014 |  |  |  | hypothetical protein |
| 42 | CDS | 516 | resA_1 |  |  | Thiol-disulfide oxidoreductase ResA |
| 43 | CDS | 420 |  |  |  | hypothetical protein |
| 44 | CDS | 702 |  |  |  | hypothetical protein |
| 45 | CDS | 2109 |  |  |  | hypothetical protein |
| 46 | CDS | 3993 | rcsC_1 | 2.7.13.3 |  | Sensor histidine kinase RcsC |
| 47 | CDS | 870 | lacX |  |  | Protein LacX, plasmid |
| 48 | CDS | 912 | cynR |  |  | HTH-type transcriptional regulator CynR |
| 49 | CDS | 486 |  | 3.1.3.48 |  | Putative low molecular weight protein-tyrosine-phosphatase |
| 50 | CDS | 984 | dnaG_2 | 2.7.7.- |  | DNA primase |
| 51 | CDS | 411 |  |  |  | hypothetical protein |
| 52 | CDS | 2370 | metE | 2.1.1.14 | COG0620 | 5-methyltetrahydropteroyltriglutamate--homocysteine methyltransferase |
| 53 | CDS | 993 |  |  |  | hypothetical protein |
| 54 | CDS | 1848 |  |  |  | hypothetical protein |
| 55 | CDS | 633 | exaE_1 |  |  | Transcriptional activator protein ExaE |
| 56 | CDS | 1527 |  |  |  | hypothetical protein |
| 57 | CDS | 1038 | emrA |  |  | Colistin resistance protein EmrA |
| 58 | CDS | 1353 |  |  |  | hypothetical protein |
| 59 | CDS | 912 | rhaS_1 |  |  | HTH-type transcriptional activator RhaS |
| 60 | CDS | 1407 | zraR |  | COG2204 | Transcriptional regulatory protein ZraR |
| 61 | CDS | 1722 |  |  |  | hypothetical protein |
| 62 | CDS | 3432 |  |  |  | hypothetical protein |
| 63 | CDS | 1299 | fucP_1 |  | COG0738 | L-fucose-proton symporter |
| 64 | CDS | 1392 | rhaB | 2.7.1.5 | COG1070 | L-Rhamnulokinase |
| 65 | CDS | 636 | otnC | 4.1.1.104 | COG0235 | 3-oxo-tetronate 4-phosphate decarboxylase |
| 66 | CDS | 1773 | fucI | 5.3.1.25 | COG2407 | L-fucose isomerase |
| 67 | CDS | 1383 |  |  |  | hypothetical protein |
| 68 | CDS | 1098 |  |  |  | hypothetical protein |
| 69 | CDS | 2283 |  |  |  | hypothetical protein |
| 70 | CDS | 453 | menI | 3.1.2.28 |  | 1,4-dihydroxy-2-naphthoyl-CoA hydrolase |
| 71 | CDS | 1047 | pchA | 5.4.4.2 | COG1169 | Salicylate biosynthesis isochorismate synthase |
| 72 | CDS | 1761 | menD | 2.2.1.9 |  | 2-succinyl-5-enolpyruvyl-6-hydroxy-3-cyclohexene-1-carboxylate synthase |
| 73 | CDS | 411 |  |  |  | hypothetical protein |
| 74 | CDS | 2175 |  |  |  | hypothetical protein |
| 75 | CDS | 591 |  |  |  | hypothetical protein |
| 76 | CDS | 756 |  |  |  | hypothetical protein |
| 77 | CDS | 1158 |  |  |  | hypothetical protein |
| 78 | CDS | 831 |  |  |  | hypothetical protein |
| 79 | CDS | 285 |  |  |  | hypothetical protein |
| 80 | CDS | 510 |  |  |  | hypothetical protein |
| 81 | CDS | 303 |  |  |  | hypothetical protein |
| 82 | CDS | 3117 | susC_1 |  |  | TonB-dependent receptor SusC |
| 83 | CDS | 1857 |  |  |  | hypothetical protein |
| 84 | CDS | 477 |  |  |  | hypothetical protein |
| 85 | CDS | 1665 | glaA | 3.2.1.- |  | Alpha-1,3-galactosidase A |
| 86 | CDS | 426 |  |  |  | hypothetical protein |
| 87 | CDS | 663 |  |  |  | hypothetical protein |
| 88 | CDS | 1437 |  |  |  | hypothetical protein |
| 89 | CDS | 585 |  |  |  | hypothetical protein |
| 90 | CDS | 813 |  |  |  | hypothetical protein |
| 91 | CDS | 1518 | nnr |  | COG0062 | Bifunctional NAD(P)H-hydrate repair enzyme Nnr |
| 92 | CDS | 840 |  | 1.10.3.- | COG1496 | Polyphenol oxidase |
| 93 | CDS | 1224 |  |  |  | hypothetical protein |
| 94 | CDS | 723 |  |  |  | hypothetical protein |
| 95 | CDS | 1023 |  |  |  | hypothetical protein |
| 96 | CDS | 255 |  |  |  | hypothetical protein |
| 97 | CDS | 777 |  |  |  | hypothetical protein |
| 98 | CDS | 915 | yfkN |  | COG0737 | Trifunctional nucleotide phosphoesterase protein YfkN |
| 99 | CDS | 2829 |  |  |  | TonB-dependent receptor P39 |
| 100 | CDS | 1023 |  |  |  | hypothetical protein |
| 101 | CDS | 2277 |  |  |  | hypothetical protein |
| 102 | CDS | 1803 |  |  |  | hypothetical protein |
| 103 | CDS | 351 |  |  |  | hypothetical protein |
| 104 | CDS | 921 |  |  |  | hypothetical protein |
| 105 | CDS | 513 |  |  |  | hypothetical protein |
| 106 | CDS | 429 |  |  |  | hypothetical protein |
| 107 | CDS | 747 |  |  |  | hypothetical protein |
| 108 | tRNA | 75 |  |  |  | tRNA-Met(cat) |
| 109 | CDS | 855 |  |  |  | hypothetical protein |
| 110 | CDS | 2802 | rcsC_2 | 2.7.13.3 |  | Sensor histidine kinase RcsC |
| 111 | CDS | 2274 |  |  |  | hypothetical protein |
| 112 | CDS | 417 |  |  |  | hypothetical protein |
| 113 | CDS | 879 |  |  |  | hypothetical protein |
| 114 | CDS | 501 |  |  |  | hypothetical protein |
| 115 | CDS | 483 | gldH |  |  | Gliding motility lipoprotein GldH |
| 116 | CDS | 1113 |  |  |  | hypothetical protein |
| 117 | CDS | 657 |  |  |  | hypothetical protein |
| 118 | CDS | 732 | rsmI | 2.1.1.198 | COG0313 | Ribosomal RNA small subunit methyltransferase I |
| 119 | CDS | 849 |  |  |  | hypothetical protein |
| 120 | CDS | 378 |  |  |  | hypothetical protein |
| 121 | CDS | 732 | birA | 6.3.4.15 | COG0340 | Bifunctional ligase/repressor BirA |
| 122 | CDS | 729 |  |  |  | hypothetical protein |
| 123 | CDS | 1614 | cheB_1 | 3.5.1.44 |  | Protein-glutamate methylesterase/protein-glutamine glutaminase |
| 124 | CDS | 1617 |  |  |  | hypothetical protein |
| 125 | CDS | 1125 |  |  |  | hypothetical protein |
| 126 | CDS | 3258 |  |  |  | hypothetical protein |
| 127 | CDS | 324 |  |  |  | hypothetical protein |
| 128 | CDS | 435 |  |  |  | hypothetical protein |
| 129 | CDS | 603 | lysO |  | COG2431 | Lysine exporter LysO |
| 130 | CDS | 384 |  |  |  | hypothetical protein |
| 131 | CDS | 228 |  |  |  | hypothetical protein |
| 132 | CDS | 1104 |  |  |  | hypothetical protein |
| 133 | CDS | 912 |  |  |  | hypothetical protein |
| 134 | CDS | 969 |  |  |  | hypothetical protein |
| 135 | CDS | 909 |  |  |  | hypothetical protein |
| 136 | CDS | 1518 |  |  |  | hypothetical protein |
| 137 | CDS | 873 |  |  |  | hypothetical protein |
| 138 | CDS | 2826 |  |  |  | hypothetical protein |
| 139 | CDS | 1224 |  |  |  | hypothetical protein |
| 140 | CDS | 1392 |  |  |  | hypothetical protein |
| 141 | CDS | 3180 |  |  |  | TonB-dependent receptor P39 |
| 142 | CDS | 3489 | lacZ_1 | 3.2.1.23 |  | Beta-galactosidase |
| 143 | CDS | 897 |  |  |  | hypothetical protein |
| 144 | CDS | 1971 |  |  |  | hypothetical protein |
| 145 | CDS | 2946 |  |  |  | TonB-dependent receptor P26 |
| 146 | CDS | 630 |  |  |  | hypothetical protein |
| 147 | CDS | 1059 |  |  |  | hypothetical protein |
| 148 | CDS | 441 |  |  |  | hypothetical protein |
| 149 | CDS | 846 |  |  |  | hypothetical protein |
| 150 | CDS | 1257 |  |  |  | hypothetical protein |
| 151 | CDS | 1287 |  |  |  | hypothetical protein |
| 152 | CDS | 1623 |  |  |  | hypothetical protein |
| 153 | CDS | 804 |  |  |  | hypothetical protein |
| 154 | CDS | 1698 |  |  |  | hypothetical protein |
| 155 | CDS | 1404 | yjmB |  | COG2211 | putative symporter YjmB |
| 156 | CDS | 831 |  |  |  | hypothetical protein |
| 157 | CDS | 843 |  |  |  | hypothetical protein |
| 158 | CDS | 2637 |  |  |  | hypothetical protein |
| 159 | CDS | 924 |  |  |  | hypothetical protein |
| 160 | tRNA | 73 |  |  |  | tRNA-Glu(ttc) |
| 161 | CDS | 726 | recO |  |  | DNA repair protein RecO |
| 162 | CDS | 1338 | ftsZ |  |  | Cell division protein FtsZ |
| 163 | CDS | 468 |  |  |  | hypothetical protein |
| 164 | CDS | 915 | rsmH | 2.1.1.199 | COG0275 | Ribosomal RNA small subunit methyltransferase H |
| 165 | CDS | 828 |  |  |  | hypothetical protein |
| 166 | CDS | 2901 |  |  |  | hypothetical protein |
| 167 | CDS | 1338 | dgt | 3.1.5.1 |  | Deoxyguanosinetriphosphate triphosphohydrolase |
| 168 | CDS | 438 | dut | 3.6.1.23 |  | Deoxyuridine 5'-triphosphate nucleotidohydrolase |
| 169 | CDS | 1866 | bepA_1 | 3.4.-.- |  | Beta-barrel assembly-enhancing protease |
| 170 | CDS | 897 |  |  |  | hypothetical protein |
| 171 | CDS | 486 |  | 3.6.1.25 | COG2954 | Inorganic triphosphatase |
| 172 | CDS | 960 |  |  |  | hypothetical protein |
| 173 | CDS | 2850 | rhaS_2 |  |  | HTH-type transcriptional activator RhaS |
| 174 | CDS | 690 |  |  |  | hypothetical protein |
| 175 | CDS | 1107 | yhcG_1 | 3.1.-.- | COG4804 | Putative nuclease YhcG |
| 176 | CDS | 687 |  |  |  | hypothetical protein |
| 177 | CDS | 1476 |  |  |  | hypothetical protein |
| 178 | CDS | 1686 |  |  |  | hypothetical protein |
| 179 | CDS | 804 | yhcG_2 | 3.1.-.- | COG4804 | Putative nuclease YhcG |
| 180 | CDS | 300 |  |  |  | hypothetical protein |
| 181 | CDS | 522 |  |  |  | hypothetical protein |
| 182 | CDS | 1128 | pgl | 3.1.1.31 |  | 6-phosphogluconolactonase |
| 183 | CDS | 486 | argR |  | COG1438 | Arginine repressor |
| 184 | CDS | 1365 |  |  |  | hypothetical protein |
| 185 | CDS | 2982 | susC_2 |  |  | TonB-dependent receptor SusC |
| 186 | CDS | 1617 | susD |  |  | Starch-binding protein SusD |
| 187 | CDS | 1785 |  |  |  | hypothetical protein |
| 188 | CDS | 624 |  |  |  | hypothetical protein |
| 189 | CDS | 2715 |  |  |  | hypothetical protein |
| 190 | CDS | 3630 |  |  |  | hypothetical protein |
| 191 | CDS | 1029 |  |  |  | hypothetical protein |
| 192 | CDS | 264 |  |  |  | hypothetical protein |
| 193 | CDS | 1998 |  |  |  | hypothetical protein |
| 194 | CDS | 543 |  |  |  | hypothetical protein |
| 195 | CDS | 768 |  |  |  | Putative bifunctional phosphatase/peptidyl-prolyl cis-trans isomerase |
| 196 | CDS | 921 |  |  |  | hypothetical protein |
| 197 | CDS | 453 |  |  |  | hypothetical protein |
| 198 | CDS | 900 | bepA_2 | 3.4.-.- | COG4783 | Beta-barrel assembly-enhancing protease |
| 199 | CDS | 1644 |  | 3.2.1.- |  | Glycosyl hydrolase family 109 protein 1 |
| 200 | CDS | 1512 |  |  |  | hypothetical protein |
| 201 | CDS | 651 |  |  |  | hypothetical protein |
| 202 | CDS | 810 |  |  |  | hypothetical protein |
| 203 | CDS | 330 |  |  |  | hypothetical protein |
| 204 | CDS | 1293 | fdtB | 2.6.1.90 |  | dTDP-3-amino-3,6-dideoxy-alpha-D-galactopyranose transaminase |
| 205 | CDS | 744 |  |  |  | hypothetical protein |
| 206 | CDS | 1527 | dnaC | 3.6.4.12 | COG0305 | Replicative DNA helicase |
| 207 | CDS | 2646 |  |  |  | hypothetical protein |
| 208 | CDS | 918 |  |  |  | hypothetical protein |
| 209 | CDS | 612 | rnd | 3.1.13.5 |  | Ribonuclease D |
| 210 | CDS | 600 |  |  |  | hypothetical protein |
| 211 | CDS | 1089 | nahK | 2.7.1.162 |  | N-acetylhexosamine 1-kinase |
| 212 | CDS | 3903 |  |  |  | hypothetical protein |
| 213 | CDS | 435 | perR_1 |  | COG0735 | Peroxide operon regulator |
| 214 | CDS | 960 |  |  |  | hypothetical protein |
| 215 | tRNA | 90 |  |  |  | tRNA-Ser(cga) |
| 216 | CDS | 585 | queE | 4.3.99.3 |  | 7-carboxy-7-deazaguanine synthase |
| 217 | CDS | 369 |  |  |  | hypothetical protein |
| 218 | CDS | 744 | kpsU | 2.7.7.38 | COG1212 | 3-deoxy-manno-octulosonate cytidylyltransferase |
| 219 | CDS | 1368 | tilS | 6.3.4.19 | COG0037 | tRNA(Ile)-lysidine synthase |
| 220 | CDS | 1719 |  |  |  | hypothetical protein |
| 221 | CDS | 873 | sigA_1 |  | COG0568 | RNA polymerase sigma factor SigA |
| 222 | CDS | 2106 |  |  |  | hypothetical protein |
| 223 | CDS | 3267 | recB | 3.1.11.5 |  | RecBCD enzyme subunit RecB |
| 224 | CDS | 2937 |  |  |  | hypothetical protein |
| 225 | CDS | 618 | hrdA |  | COG0568 | RNA polymerase principal sigma factor HrdA |
| 226 | CDS | 555 |  |  |  | hypothetical protein |
| 227 | CDS | 1908 |  |  |  | hypothetical protein |
| 228 | CDS | 690 |  |  |  | hypothetical protein |
| 229 | CDS | 393 |  |  |  | hypothetical protein |
| 230 | CDS | 642 |  |  |  | hypothetical protein |
| 231 | CDS | 1071 | manC1 | 2.7.7.13 | COG0662 | Mannose-1-phosphate guanylyltransferase 1 |
| 232 | CDS | 345 |  |  |  | hypothetical protein |
| 233 | CDS | 504 |  |  |  | hypothetical protein |
| 234 | CDS | 432 |  |  |  | hypothetical protein |
| 235 | CDS | 546 | sigW |  | COG1595 | ECF RNA polymerase sigma factor SigW |
| 236 | CDS | 1536 |  |  |  | hypothetical protein |
| 237 | CDS | 318 |  |  |  | hypothetical protein |
| 238 | CDS | 1989 | fbp | 3.1.3.11 | COG3855 | Fructose-1,6-bisphosphatase class 3 |
| 239 | CDS | 897 |  |  |  | hypothetical protein |
| 240 | CDS | 627 |  |  |  | hypothetical protein |
| 241 | CDS | 2418 | btuB_1 |  |  | Vitamin B12 transporter BtuB |
| 242 | CDS | 906 |  |  |  | hypothetical protein |
| 243 | CDS | 2034 | rlhA |  | COG0826 | 23S rRNA 5-hydroxycytidine synthase |
| 244 | CDS | 918 | metAA | 2.3.1.31 | COG1897 | Homoserine O-acetyltransferase |
| 245 | CDS | 543 |  |  |  | hypothetical protein |
| 246 | CDS | 1491 |  |  |  | hypothetical protein |
| 247 | CDS | 444 |  |  |  | hypothetical protein |
| 248 | CDS | 348 |  |  |  | hypothetical protein |
| 249 | CDS | 1320 | dbpA | 3.6.4.13 | COG0513 | ATP-dependent RNA helicase DbpA |
| 250 | CDS | 1254 |  |  |  | hypothetical protein |
| 251 | CDS | 444 |  |  | COG2731 | putative protein |
| 252 | CDS | 711 | btsR |  | COG3279 | Transcriptional regulatory protein BtsR |
| 253 | CDS | 1437 | gph_1 | 3.1.3.18 |  | Phosphoglycolate phosphatase |
| 254 | CDS | 951 | rhgT | 3.1.1.- | COG2755 | Rhamnogalacturonan acetylesterase RhgT |
| 255 | CDS | 465 | perR_2 |  | COG0735 | Peroxide operon regulator |
| 256 | CDS | 663 | yhdE | 3.6.1.9 | COG0424 | dTTP/UTP pyrophosphatase |
| 257 | CDS | 504 | kdsC | 3.1.3.45 | COG1778 | 3-deoxy-D-manno-octulosonate 8-phosphate phosphatase KdsC |
| 258 | CDS | 777 |  |  |  | hypothetical protein |
| 259 | CDS | 1011 |  |  |  | hypothetical protein |
| 260 | CDS | 621 |  |  |  | hypothetical protein |
| 261 | CDS | 615 |  |  |  | hypothetical protein |
| 262 | CDS | 1311 |  |  |  | Outer membrane protein 40 |
| 263 | CDS | 1434 |  |  |  | hypothetical protein |
| 264 | CDS | 906 |  |  |  | hypothetical protein |
| 265 | CDS | 498 |  |  |  | hypothetical protein |
| 266 | CDS | 357 |  |  |  | hypothetical protein |
| 267 | CDS | 291 |  |  |  | hypothetical protein |
| 268 | CDS | 456 |  |  |  | hypothetical protein |
| 269 | CDS | 1179 |  |  |  | hypothetical protein |
| 270 | CDS | 630 |  |  |  | hypothetical protein |
| 271 | CDS | 1053 |  |  |  | hypothetical protein |
| 272 | CDS | 765 | tcdA | 6.1.-.- | COG1179 | tRNA threonylcarbamoyladenosine dehydratase |
| 273 | CDS | 2235 | recD2_2 | 3.6.4.12 | COG0507 | ATP-dependent RecD-like DNA helicase |
| 274 | CDS | 219 |  |  |  | hypothetical protein |
| 275 | CDS | 1215 | nagA | 3.2.1.49 |  | Alpha-N-acetylgalactosaminidase |
| 276 | CDS | 1746 | ihfA |  |  | Integration host factor subunit alpha |
| 277 | CDS | 294 | hup |  |  | DNA-binding protein HU |
| 278 | CDS | 954 | ftsY |  | COG0552 | Signal recognition particle receptor FtsY |
| 279 | CDS | 954 |  |  |  | hypothetical protein |
| 280 | CDS | 339 |  |  |  | hypothetical protein |
| 281 | CDS | 921 | menA | 2.5.1.74 |  | 1,4-dihydroxy-2-naphthoate octaprenyltransferase |
| 282 | CDS | 642 |  |  |  | hypothetical protein |
| 283 | tRNA | 89 |  |  |  | tRNA-Ser(gct) |
| 284 | tRNA | 74 |  |  |  | tRNA-Glu(ctc) |
| 285 | tRNA | 74 |  |  |  | tRNA-Glu(ctc) |
| 286 | tRNA | 73 |  |  |  | tRNA-Glu(ctc) |
| 287 | tRNA | 88 |  |  |  | tRNA-Ser(gct) |
| 288 | tRNA | 73 |  |  |  | tRNA-Glu(ctc) |
| 289 | CDS | 1269 |  |  |  | hypothetical protein |
| 290 | CDS | 435 |  |  |  | hypothetical protein |
| 291 | CDS | 354 |  |  |  | hypothetical protein |
| 292 | CDS | 1125 | aroC | 4.2.3.5 |  | Chorismate synthase |
| 293 | CDS | 648 | nth | 4.2.99.18 | COG0177 | Endonuclease III |
| 294 | CDS | 384 |  |  |  | hypothetical protein |
| 295 | CDS | 546 |  |  |  | hypothetical protein |
| 296 | CDS | 1221 |  |  |  | hypothetical protein |
| 297 | CDS | 2172 |  |  |  | hypothetical protein |
| 298 | CDS | 510 | speG | 2.3.1.57 |  | Spermidine N(1)-acetyltransferase |
| 299 | CDS | 1152 |  |  |  | hypothetical protein |
| 300 | CDS | 612 | recR |  | COG0353 | Recombination protein RecR |
| 301 | CDS | 483 |  |  |  | hypothetical protein |
| 302 | CDS | 1545 | cstA |  | COG1966 | Peptide transporter CstA |
| 303 | CDS | 1725 |  |  |  | hypothetical protein |
| 304 | tRNA | 74 |  |  |  | tRNA-Lys(ctt) |
| 305 | tRNA | 74 |  |  |  | tRNA-Lys(ctt) |
| 306 | tRNA | 74 |  |  |  | tRNA-Lys(ctt) |
| 307 | CDS | 423 |  |  |  | hypothetical protein |
| 308 | CDS | 1365 |  |  |  | hypothetical protein |
| 309 | CDS | 1014 |  |  |  | IS1595 family transposase ISCco3 |
| 310 | CDS | 903 |  |  |  | hypothetical protein |
| 311 | CDS | 495 |  |  |  | hypothetical protein |
| 312 | CDS | 1635 |  |  |  | hypothetical protein |
| 313 | CDS | 873 | prmA | 2.1.1.- | COG2264 | Ribosomal protein L11 methyltransferase |
| 314 | CDS | 273 |  |  |  | hypothetical protein |
| 315 | CDS | 558 |  |  |  | hypothetical protein |
| 316 | CDS | 1035 | hisC | 2.6.1.9 | COG0079 | Histidinol-phosphate aminotransferase |
| 317 | CDS | 1116 | resA_2 |  |  | Thiol-disulfide oxidoreductase ResA |
| 318 | CDS | 807 |  |  |  | hypothetical protein |
| 319 | CDS | 555 | rfbC | 5.1.3.13 | COG1898 | dTDP-4-dehydrorhamnose 3,5-epimerase |
| 320 | CDS | 1101 |  |  |  | hypothetical protein |
| 321 | CDS | 534 |  |  |  | hypothetical protein |
| 322 | CDS | 717 | hisA | 5.3.1.16 | COG0106 | 1-(5-phosphoribosyl)-5-[(5-phosphoribosylamino)methylideneamino] imidazole-4-carboxamide isomerase |
| 323 | CDS | 780 | hisF | 4.3.2.10 | COG0107 | Imidazole glycerol phosphate synthase subunit HisF |
| 324 | CDS | 648 | hisI | 3.5.4.19 |  | Histidine biosynthesis bifunctional protein HisIE |
| 325 | CDS | 774 |  |  |  | hypothetical protein |
| 326 | CDS | 198 | thiS |  | COG2104 | Sulfur carrier protein ThiS |
| 327 | CDS | 651 | thiE_1 | 2.5.1.3 | COG0352 | Thiamine-phosphate synthase |
| 328 | CDS | 681 | mprA_1 |  |  | Response regulator MprA |
| 329 | CDS | 1359 | sasA_1 | 2.7.-.- |  | Adaptive-response sensory-kinase SasA |
| 330 | CDS | 1101 |  |  |  | hypothetical protein |
| 331 | CDS | 1038 |  |  |  | hypothetical protein |
| 332 | CDS | 1137 | hutG | 3.5.3.8 |  | Formimidoylglutamase |
| 333 | CDS | 2202 | ltxB | 7.4.2.5 | COG2274 | Leukotoxin export ATP-binding protein LtxB |
| 334 | CDS | 2073 |  |  |  | hypothetical protein |
| 335 | CDS | 954 | gspA |  | COG1442 | General stress protein A |
| 336 | CDS | 1035 | wbbI | 2.4.1.- |  | Beta-1,6-galactofuranosyltransferase WbbI |
| 337 | CDS | 1263 | pknD | 2.7.11.1 |  | Serine/threonine-protein kinase PknD |
| 338 | CDS | 1734 |  |  |  | hypothetical protein |
| 339 | CDS | 570 |  |  |  | hypothetical protein |
| 340 | CDS | 1386 |  |  |  | hypothetical protein |
| 341 | CDS | 669 |  |  |  | hypothetical protein |
| 342 | CDS | 753 | ispU | 2.5.1.31 | COG0020 | Ditrans,polycis-undecaprenyl-diphosphate synthase ((2E,6E)-farnesyl-diphosphate specific) |
| 343 | CDS | 837 | murI | 5.1.1.3 | COG0796 | Glutamate racemase |
| 344 | CDS | 1230 | lolE |  | COG4591 | Lipoprotein-releasing system transmembrane protein LolE |
| 345 | CDS | 639 | trmR | 2.1.1.- |  | tRNA 5-hydroxyuridine methyltransferase |
| 346 | CDS | 435 | aroQ | 4.2.1.10 | COG0757 | 3-dehydroquinate dehydratase |
| 347 | CDS | 906 | xerD |  |  | Tyrosine recombinase XerD |
| 348 | CDS | 750 |  |  |  | hypothetical protein |
| 349 | CDS | 405 | rnpA | 3.1.26.5 |  | Ribonuclease P protein component |
| 350 | tRNA | 72 |  |  |  | tRNA-Gln(ttg) |
| 351 | CDS | 834 |  |  |  | hypothetical protein |
| 352 | CDS | 276 | hupB |  | COG0776 | DNA-binding protein HU-beta |
| 353 | CDS | 975 |  |  |  | hypothetical protein |
| 354 | CDS | 1098 |  |  |  | hypothetical protein |
| 355 | tRNA | 73 |  |  |  | tRNA-Arg(cct) |
| 356 | CDS | 957 |  |  |  | hypothetical protein |
| 357 | CDS | 1161 |  |  |  | hypothetical protein |
| 358 | CDS | 1224 | pal_1 |  |  | Peptidoglycan-associated lipoprotein |
| 359 | CDS | 1074 | yhcG_3 | 3.1.-.- | COG4804 | Putative nuclease YhcG |
| 360 | CDS | 738 |  |  |  | hypothetical protein |
| 361 | CDS | 1008 |  |  |  | hypothetical protein |
| 362 | tRNA | 87 |  |  |  | tRNA-Leu(taa) |
| 363 | CDS | 615 |  |  |  | hypothetical protein |
| 364 | CDS | 729 |  |  |  | hypothetical protein |
| 365 | CDS | 1038 | ribD |  | COG0117 | Riboflavin biosynthesis protein RibD |
| 366 | CDS | 3294 | lacZ_2 | 3.2.1.23 |  | Beta-galactosidase |
| 367 | CDS | 1365 |  |  |  | hypothetical protein |
| 368 | CDS | 870 | RBKS | 2.7.1.15 |  | Ribokinase |
| 369 | CDS | 1764 |  |  |  | hypothetical protein |
| 370 | CDS | 900 |  |  |  | hypothetical protein |
| 371 | CDS | 798 |  |  |  | hypothetical protein |
| 372 | CDS | 699 | ompR |  |  | Transcriptional regulatory protein OmpR |
| 373 | CDS | 1191 | hemW |  | COG0635 | Heme chaperone HemW |
| 374 | CDS | 363 |  |  |  | hypothetical protein |
| 375 | CDS | 843 |  |  |  | hypothetical protein |
| 376 | CDS | 1803 | deaD | 3.6.4.13 |  | ATP-dependent RNA helicase DeaD |
| 377 | CDS | 843 |  |  |  | hypothetical protein |
| 378 | CDS | 2682 |  |  |  | hypothetical protein |
| 379 | CDS | 666 |  |  |  | hypothetical protein |
| 380 | CDS | 273 |  |  |  | hypothetical protein |
| 381 | CDS | 909 | pyrD | 1.3.1.14 | COG0167 | Dihydroorotate dehydrogenase B (NAD(+)), catalytic subunit |
| 382 | CDS | 651 |  |  |  | hypothetical protein |
| 383 | CDS | 1029 | yqeN |  | COG1466 | putative protein YqeN |
| 384 | CDS | 429 |  |  |  | hypothetical protein |
| 385 | CDS | 426 |  |  |  | hypothetical protein |
| 386 | CDS | 492 |  |  |  | hypothetical protein |
| 387 | CDS | 525 |  |  |  | hypothetical protein |
| 388 | CDS | 435 |  |  |  | hypothetical protein |
| 389 | CDS | 876 |  | 3.5.1.77 |  | N-carbamoyl-D-amino acid hydrolase |
| 390 | tRNA | 75 |  |  |  | tRNA-Arg(ccg) |
| 391 | CDS | 981 |  |  |  | hypothetical protein |
| 392 | tmRNA | 397 | ssrA |  |  | transfer-messenger RNA, SsrA |
| 393 | CDS | 747 |  |  |  | hypothetical protein |
| 394 | CDS | 585 |  |  |  | hypothetical protein |
| 395 | CDS | 1659 |  |  |  | hypothetical protein |
| 396 | CDS | 3258 |  |  |  | hypothetical protein |
| 397 | CDS | 828 | rhaS_3 |  |  | HTH-type transcriptional activator RhaS |
| 398 | CDS | 837 |  | 3.2.1.103 |  | Keratan-sulfate endo-1,4-beta-galactosidase |
| 399 | CDS | 435 |  |  |  | hypothetical protein |
| 400 | CDS | 2457 |  |  |  | hypothetical protein |
| 401 | CDS | 2094 |  |  |  | hypothetical protein |
| 402 | CDS | 3186 | mdtC_1 |  | COG0841 | Multidrug resistance protein MdtC |
| 403 | CDS | 1098 | srpA |  |  | Solvent efflux pump periplasmic linker SrpA |
| 404 | CDS | 1221 | agaA | 3.2.1.22 |  | Alpha-galactosidase A |
| 405 | CDS | 945 |  |  |  | hypothetical protein |
| 406 | CDS | 600 |  |  |  | hypothetical protein |
| 407 | CDS | 726 |  |  |  | hypothetical protein |
| 408 | CDS | 1065 | purR |  |  | HTH-type transcriptional repressor PurR |
| 409 | CDS | 1311 | mleN |  | COG1757 | Malate-2H(+)/Na(+)-lactate antiporter |
| 410 | CDS | 696 |  |  |  | hypothetical protein |
| 411 | CDS | 687 | acm | 3.2.1.17 |  | Lysozyme M1 |
| 412 | CDS | 1269 |  |  |  | hypothetical protein |
| 413 | CDS | 1179 | cntE |  |  | Staphylopine export protein |
| 414 | CDS | 444 |  |  |  | hypothetical protein |
| 415 | CDS | 1122 | dnaN |  | COG0592 | Beta sliding clamp |
| 416 | CDS | 924 |  |  |  | hypothetical protein |
| 417 | CDS | 1626 |  |  |  | hypothetical protein |
| 418 | tRNA | 76 |  |  |  | tRNA-Arg(tct) |
| 419 | CDS | 3915 | lacZ_3 | 3.2.1.23 |  | Beta-galactosidase |
| 420 | CDS | 1155 | fucO | 1.1.1.77 | COG1454 | Lactaldehyde reductase |
| 421 | CDS | 450 |  |  |  | hypothetical protein |
| 422 | CDS | 1584 |  |  |  | hypothetical protein |
| 423 | CDS | 1050 |  |  |  | hypothetical protein |
| 424 | CDS | 1554 |  |  |  | hypothetical protein |
| 425 | CDS | 1056 |  |  |  | hypothetical protein |
| 426 | CDS | 1152 |  |  |  | hypothetical protein |
| 427 | CDS | 816 |  |  |  | hypothetical protein |
| 428 | CDS | 822 |  |  |  | hypothetical protein |
| 429 | CDS | 810 | tuaG | 2.4.-.- | COG0463 | Putative teichuronic acid biosynthesis glycosyltransferase TuaG |
| 430 | CDS | 636 | wecA_1 | 2.7.8.40 |  | UDP-N-acetylgalactosamine-undecaprenyl-phosphate N-acetylgalactosaminephosphotransferase |
| 431 | CDS | 1275 |  |  |  | hypothetical protein |
| 432 | CDS | 3192 | czcA |  |  | Cobalt-zinc-cadmium resistance protein CzcA |
| 433 | CDS | 1248 | czcB |  |  | Cobalt-zinc-cadmium resistance protein CzcB |
| 434 | CDS | 2076 | dsbD | 1.8.1.8 | COG4232 | Thiol:disulfide interchange protein DsbD |
| 435 | CDS | 1290 |  |  |  | hypothetical protein |
| 436 | CDS | 1068 |  |  |  | hypothetical protein |
| 437 | CDS | 2619 |  |  |  | hypothetical protein |
| 438 | CDS | 1584 |  |  |  | hypothetical protein |
| 439 | CDS | 546 | yfcE | 3.1.4.- | COG0622 | Phosphodiesterase YfcE |
| 440 | CDS | 1251 | apgM |  |  | 2,3-bisphosphoglycerate-independent phosphoglycerate mutase |
| 441 | CDS | 681 |  |  |  | hypothetical protein |
| 442 | CDS | 957 | hpr | 1.1.1.81 | COG1052 | Hydroxypyruvate reductase |
| 443 | CDS | 1041 | btuC |  | COG4139 | Vitamin B12 import system permease protein BtuC |
| 444 | CDS | 1209 |  |  |  | hypothetical protein |
| 445 | CDS | 2835 | clpX |  |  | ATP-dependent Clp protease ATP-binding subunit ClpX |
| 446 | tRNA | 74 |  |  |  | tRNA-Gly(ccc) |
| 447 | CDS | 408 |  |  |  | hypothetical protein |
| 448 | CDS | 867 |  |  |  | hypothetical protein |
| 449 | CDS | 1254 | fpgS | 6.3.2.17 |  | Folylpolyglutamate synthase |
| 450 | CDS | 1347 |  |  |  | hypothetical protein |
| 451 | CDS | 609 |  |  |  | hypothetical protein |
| 452 | CDS | 645 |  |  |  | hypothetical protein |
| 453 | CDS | 1428 |  |  |  | hypothetical protein |
| 454 | CDS | 771 | cutC |  | COG3142 | Copper homeostasis protein CutC |
| 455 | CDS | 2667 |  |  |  | hypothetical protein |
| 456 | CDS | 873 |  |  |  | hypothetical protein |
| 457 | CDS | 2559 |  |  |  | hypothetical protein |
| 458 | CDS | 2073 |  |  |  | hypothetical protein |
| 459 | CDS | 687 |  |  |  | hypothetical protein |
| 460 | CDS | 1029 | sdsA | 2.5.1.84 |  | All-trans-nonaprenyl-diphosphate synthase (geranyl-diphosphate specific) |
| 461 | CDS | 939 | deoC | 4.1.2.4 |  | Deoxyribose-phosphate aldolase |
| 462 | CDS | 351 | ypjD |  | COG1694 | putative protein YpjD |
| 463 | CDS | 1152 | yghO_1 |  |  | Protein YghO |
| 464 | CDS | 441 |  |  |  | hypothetical protein |
| 465 | CDS | 471 |  |  |  | hypothetical protein |
| 466 | CDS | 1314 |  |  |  | hypothetical protein |
| 467 | CDS | 738 |  |  |  | hypothetical protein |
| 468 | tRNA | 75 |  |  |  | tRNA-Met(cat) |
| 469 | CDS | 3138 | nanM | 5.1.3.24 |  | N-acetylneuraminate epimerase |
| 470 | CDS | 1866 |  |  |  | hypothetical protein |
| 471 | CDS | 1803 |  |  |  | hypothetical protein |
| 472 | CDS | 3243 |  |  |  | hypothetical protein |
| 473 | CDS | 396 |  |  |  | hypothetical protein |
| 474 | CDS | 1278 |  |  |  | hypothetical protein |
| 475 | CDS | 1188 | ce | 5.1.3.11 |  | Cellobiose 2-epimerase |
| 476 | CDS | 1236 | mdtG |  |  | Multidrug resistance protein MdtG |
| 477 | CDS | 1197 | mlc |  | COG1940 | Protein mlc |
| 478 | CDS | 792 | nagB | 3.5.99.6 | COG0363 | Glucosamine-6-phosphate deaminase |
| 479 | CDS | 2640 | lacZ_4 | 3.2.1.23 |  | Beta-galactosidase |
| 480 | CDS | 1560 |  |  |  | SusD-like protein P2 |
| 481 | CDS | 3087 |  |  |  | TonB-dependent receptor P39 |
| 482 | CDS | 3870 | rcsC_3 | 2.7.13.3 |  | Sensor histidine kinase RcsC |
| 483 | CDS | 873 |  |  |  | hypothetical protein |
| 484 | CDS | 1401 |  |  |  | hypothetical protein |
| 485 | CDS | 1107 | ald | 1.4.1.1 | COG0686 | Alanine dehydrogenase |
| 486 | CDS | 1254 |  |  |  | hypothetical protein |
| 487 | CDS | 2022 |  |  |  | hypothetical protein |
| 488 | CDS | 3183 |  |  |  | TonB-dependent receptor P3 |
| 489 | CDS | 366 | mcrB | 3.1.21.- | COG1401 | 5-methylcytosine-specific restriction enzyme B |
| 490 | CDS | 1056 | mcrC |  | COG4268 | Protein McrC |
| 491 | CDS | 756 |  |  |  | hypothetical protein |
| 492 | CDS | 1185 |  |  |  | hypothetical protein |
| 493 | CDS | 699 |  |  |  | hypothetical protein |
| 494 | CDS | 495 | lrp |  | COG1522 | Leucine-responsive regulatory protein |
| 495 | CDS | 603 |  | 3.4.21.- | COG3340 | putative peptidase |
| 496 | CDS | 819 |  |  |  | hypothetical protein |
| 497 | CDS | 2265 |  |  |  | hypothetical protein |
| 498 | CDS | 1359 |  |  |  | hypothetical protein |
| 499 | CDS | 1728 | recF |  |  | DNA replication and repair protein RecF |
| 500 | CDS | 864 |  |  |  | hypothetical protein |
| 501 | CDS | 558 |  |  |  | hypothetical protein |
| 502 | CDS | 1071 |  |  |  | hypothetical protein |
| 503 | CDS | 4137 |  |  |  | hypothetical protein |
| 504 | CDS | 1008 |  |  |  | hypothetical protein |
| 505 | CDS | 1536 |  |  |  | hypothetical protein |
| 506 | CDS | 1380 |  |  |  | hypothetical protein |
| 507 | CDS | 1608 | exoI | 3.2.1.52 | COG3525 | Beta-hexosaminidase |
| 508 | CDS | 1665 |  |  |  | hypothetical protein |
| 509 | CDS | 951 |  |  |  | hypothetical protein |
| 510 | CDS | 1212 |  |  |  | hypothetical protein |
| 511 | CDS | 1257 |  |  |  | hypothetical protein |
| 512 | CDS | 420 |  |  |  | hypothetical protein |
| 513 | CDS | 1203 |  |  |  | hypothetical protein |
| 514 | CDS | 2820 |  |  |  | hypothetical protein |
| 515 | CDS | 2826 |  |  |  | hypothetical protein |
| 516 | CDS | 825 |  |  |  | hypothetical protein |
| 517 | CDS | 630 |  |  |  | hypothetical protein |
| 518 | CDS | 570 |  |  |  | hypothetical protein |
| 519 | CDS | 327 |  |  |  | hypothetical protein |
| 520 | CDS | 816 |  |  |  | hypothetical protein |
| 521 | CDS | 1386 |  |  |  | hypothetical protein |
| 522 | CDS | 894 |  |  |  | hypothetical protein |
| 523 | CDS | 951 | arnC | 2.4.2.53 |  | Undecaprenyl-phosphate 4-deoxy-4-formamido-L-arabinose transferase |
| 524 | CDS | 1191 |  |  |  | hypothetical protein |
| 525 | CDS | 984 |  |  |  | hypothetical protein |
| 526 | CDS | 1254 |  |  |  | hypothetical protein |
| 527 | CDS | 1533 | epsK |  |  | putative membrane protein EpsK |
| 528 | CDS | 714 |  |  |  | hypothetical protein |
| 529 | CDS | 1992 |  |  |  | hypothetical protein |
| 530 | CDS | 636 |  |  |  | hypothetical protein |
| 531 | CDS | 1014 |  |  |  | hypothetical protein |
| 532 | CDS | 375 |  |  |  | hypothetical protein |
| 533 | CDS | 438 | fucP_2 |  | COG0738 | L-fucose-proton symporter |
| 534 | CDS | 930 |  |  |  | hypothetical protein |
| 535 | CDS | 1470 |  |  |  | hypothetical protein |
| 536 | CDS | 690 | yiaD |  | COG2885 | putative lipoprotein YiaD |
| 537 | CDS | 369 |  |  |  | hypothetical protein |
| 538 | CDS | 954 |  |  |  | hypothetical protein |
| 539 | CDS | 1533 |  |  |  | hypothetical protein |
| 540 | CDS | 768 | rsmE | 2.1.1.193 | COG1385 | Ribosomal RNA small subunit methyltransferase E |
| 541 | CDS | 570 |  |  |  | hypothetical protein |
| 542 | CDS | 648 |  |  |  | hypothetical protein |
| 543 | CDS | 357 |  |  |  | hypothetical protein |
| 544 | CDS | 429 | glpE | 2.8.1.1 |  | Thiosulfate sulfurtransferase GlpE |
| 545 | CDS | 1134 |  |  |  | hypothetical protein |
| 546 | CDS | 675 |  |  |  | hypothetical protein |
| 547 | CDS | 771 |  |  |  | hypothetical protein |
| 548 | CDS | 1407 |  |  |  | hypothetical protein |
| 549 | CDS | 321 |  |  |  | hypothetical protein |
| 550 | CDS | 1848 |  |  |  | hypothetical protein |
| 551 | CDS | 1179 |  |  |  | hypothetical protein |
| 552 | CDS | 447 |  |  |  | hypothetical protein |
| 553 | CDS | 480 |  |  |  | hypothetical protein |
| 554 | CDS | 1029 | dnaG_3 | 2.7.7.- |  | DNA primase |
| 555 | CDS | 1446 |  |  |  | hypothetical protein |
| 556 | CDS | 630 |  |  |  | hypothetical protein |
| 557 | CDS | 1587 |  |  |  | hypothetical protein |
| 558 | CDS | 819 | cpdA_1 | 3.1.4.53 |  | 3',5'-cyclic adenosine monophosphate phosphodiesterase CpdA |
| 559 | CDS | 426 |  |  |  | hypothetical protein |
| 560 | tRNA | 74 |  |  |  | tRNA-Lys(ctt) |
| 561 | CDS | 369 |  |  |  | hypothetical protein |
| 562 | CDS | 480 |  |  |  | hypothetical protein |
| 563 | CDS | 711 |  |  |  | hypothetical protein |
| 564 | CDS | 294 |  |  |  | hypothetical protein |
| 565 | CDS | 2304 |  |  |  | hypothetical protein |
| 566 | CDS | 2595 |  |  |  | hypothetical protein |
| 567 | CDS | 612 |  |  |  | hypothetical protein |
| 568 | CDS | 492 |  |  |  | hypothetical protein |
| 569 | CDS | 1587 |  |  |  | hypothetical protein |
| 570 | CDS | 594 |  |  |  | hypothetical protein |
| 571 | CDS | 411 | ssb_1 |  | COG0629 | Single-stranded DNA-binding protein |
| 572 | tRNA | 87 |  |  |  | tRNA-Leu(gag) |
| 573 | tRNA | 85 |  |  |  | tRNA-Leu(gag) |
| 574 | CDS | 1176 | nspC_1 | 4.1.1.96 |  | Carboxynorspermidine/carboxyspermidine decarboxylase |
| 575 | CDS | 396 |  |  |  | hypothetical protein |
| 576 | CDS | 411 | ssb_2 |  | COG0629 | Single-stranded DNA-binding protein |
| 577 | tRNA | 87 |  |  |  | tRNA-Leu(gag) |
| 578 | tRNA | 85 |  |  |  | tRNA-Leu(gag) |
| 579 | CDS | 1176 | nspC_2 | 4.1.1.96 |  | Carboxynorspermidine/carboxyspermidine decarboxylase |
| 580 | CDS | 396 |  |  |  | hypothetical protein |
| 581 | CDS | 1152 |  |  |  | hypothetical protein |
| 582 | CDS | 357 |  |  |  | hypothetical protein |
| 583 | CDS | 1395 | allB | 3.5.2.5 | COG0044 | Allantoinase |
| 584 | CDS | 759 |  |  |  | hypothetical protein |
| 585 | CDS | 3447 | mfd | 3.6.4.- |  | Transcription-repair-coupling factor |
| 586 | CDS | 4056 | rhaS_4 |  |  | HTH-type transcriptional activator RhaS |
| 587 | CDS | 729 |  |  |  | hypothetical protein |
| 588 | CDS | 1053 |  |  |  | hypothetical protein |
| 589 | CDS | 576 |  |  |  | hypothetical protein |
| 590 | CDS | 624 | udk | 2.7.1.48 | COG0572 | Uridine kinase |
| 591 | CDS | 606 |  |  |  | hypothetical protein |
| 592 | CDS | 579 | gpmB | 5.4.2.- |  | phosphoglycerate mutase GpmB |
| 593 | CDS | 753 | ydfG | 1.1.1.381 | COG4221 | NADP-dependent 3-hydroxy acid dehydrogenase YdfG |
| 594 | CDS | 1617 |  |  |  | hypothetical protein |
| 595 | CDS | 963 |  |  |  | hypothetical protein |
| 596 | CDS | 1170 |  |  |  | hypothetical protein |
| 597 | CDS | 1731 |  |  |  | hypothetical protein |
| 598 | CDS | 969 |  |  |  | hypothetical protein |
| 599 | CDS | 2205 | hppA1 | 7.2.3.- | COG3808 | Putative K(+)-stimulated pyrophosphate-energized sodium pump |
| 600 | CDS | 1110 |  |  |  | hypothetical protein |
| 601 | CDS | 774 |  |  |  | hypothetical protein |
| 602 | CDS | 753 |  |  |  | hypothetical protein |
| 603 | CDS | 1137 | cpdA_2 | 3.1.4.53 |  | 3',5'-cyclic adenosine monophosphate phosphodiesterase CpdA |
| 604 | CDS | 600 |  |  |  | hypothetical protein |
| 605 | CDS | 984 | xerC_1 |  |  | Tyrosine recombinase XerC |
| 606 | CDS | 1305 |  |  |  | hypothetical protein |
| 607 | CDS | 723 |  |  |  | hypothetical protein |
| 608 | CDS | 516 |  |  |  | hypothetical protein |
| 609 | CDS | 717 |  |  |  | hypothetical protein |
| 610 | CDS | 1494 |  |  |  | hypothetical protein |
| 611 | CDS | 933 |  |  |  | hypothetical protein |
| 612 | CDS | 414 | fdtA | 5.3.2.3 |  | TDP-4-oxo-6-deoxy-alpha-D-glucose-3,4-oxoisomerase |
| 613 | CDS | 2079 | pal_2 |  |  | Peptidoglycan-associated lipoprotein |
| 614 | CDS | 465 |  |  |  | hypothetical protein |
| 615 | CDS | 1428 | rsmF | 2.1.1.- | COG0144 | Ribosomal RNA small subunit methyltransferase F |
| 616 | CDS | 522 | dfrA | 1.5.1.3 | COG0262 | Dihydrofolate reductase |
| 617 | CDS | 465 |  |  |  | hypothetical protein |
| 618 | CDS | 600 |  |  |  | hypothetical protein |
| 619 | CDS | 714 | tolQ |  |  | Tol-Pal system protein TolQ |
| 620 | CDS | 2538 |  |  |  | hypothetical protein |
| 621 | CDS | 738 |  |  |  | hypothetical protein |
| 622 | CDS | 948 |  |  |  | hypothetical protein |
| 623 | CDS | 693 | cmk | 2.7.4.25 | COG0283 | Cytidylate kinase |
| 624 | CDS | 420 |  |  |  | hypothetical protein |
| 625 | CDS | 699 | ywqE | 3.1.3.48 | COG4464 | Tyrosine-protein phosphatase YwqE |
| 626 | CDS | 2505 |  |  |  | hypothetical protein |
| 627 | CDS | 801 |  |  |  | hypothetical protein |
| 628 | CDS | 1431 |  |  |  | hypothetical protein |
| 629 | CDS | 1422 | dnaA |  | COG0593 | Chromosomal replication initiator protein DnaA |
| 630 | CDS | 900 |  |  |  | hypothetical protein |
| 631 | CDS | 384 | folB | 4.1.2.25 | COG1539 | Dihydroneopterin aldolase |
| 632 | CDS | 696 |  |  |  | hypothetical protein |
| 633 | CDS | 453 |  |  |  | hypothetical protein |
| 634 | CDS | 255 |  |  |  | hypothetical protein |
| 635 | CDS | 441 |  |  |  | hypothetical protein |
| 636 | CDS | 1494 |  |  |  | hypothetical protein |
| 637 | CDS | 1128 |  |  |  | hypothetical protein |
| 638 | CDS | 1251 |  |  |  | hypothetical protein |
| 639 | CDS | 2793 |  |  |  | hypothetical protein |
| 640 | CDS | 1260 |  |  |  | hypothetical protein |
| 641 | CDS | 804 |  |  |  | hypothetical protein |
| 642 | CDS | 489 |  |  |  | hypothetical protein |
| 643 | CDS | 582 | rlpA | 4.2.2.- |  | Endolytic peptidoglycan transglycosylase RlpA |
| 644 | CDS | 807 |  |  |  | hypothetical protein |
| 645 | CDS | 801 | map | 3.4.11.18 | COG0024 | Methionine aminopeptidase 1 |
| 646 | CDS | 642 |  |  |  | hypothetical protein |
| 647 | CDS | 516 |  |  |  | hypothetical protein |
| 648 | CDS | 519 | rplJ |  | COG0244 | 50S ribosomal protein L10 |
| 649 | tRNA | 73 |  |  |  | tRNA-Thr(ggt) |
| 650 | tRNA | 74 |  |  |  | tRNA-Gly(tcc) |
| 651 | tRNA | 83 |  |  |  | tRNA-Tyr(gta) |
| 652 | tRNA | 75 |  |  |  | tRNA-Thr(tgt) |
| 653 | CDS | 885 | xerC_2 |  | COG4973 | Tyrosine recombinase XerC |
| 654 | CDS | 1788 |  |  |  | hypothetical protein |
| 655 | CDS | 636 |  |  |  | hypothetical protein |
| 656 | CDS | 1044 |  |  |  | hypothetical protein |
| 657 | CDS | 726 |  |  |  | hypothetical protein |
| 658 | CDS | 936 |  |  |  | hypothetical protein |
| 659 | CDS | 582 |  |  |  | hypothetical protein |
| 660 | CDS | 1296 | moaA_1 |  |  | GTP 3',8-cyclase |
| 661 | CDS | 2109 |  |  |  | hypothetical protein |
| 662 | CDS | 672 |  |  |  | hypothetical protein |
| 663 | CDS | 1119 |  |  |  | hypothetical protein |
| 664 | CDS | 2277 |  |  |  | hypothetical protein |
| 665 | CDS | 1377 | sasA_2 | 2.7.-.- |  | Adaptive-response sensory-kinase SasA |
| 666 | CDS | 681 | mprA_2 |  |  | Response regulator MprA |
| 667 | CDS | 1179 |  |  |  | hypothetical protein |
| 668 | CDS | 1065 |  |  |  | hypothetical protein |
| 669 | CDS | 2025 |  |  |  | hypothetical protein |
| 670 | CDS | 951 |  |  |  | hypothetical protein |
| 671 | CDS | 1752 |  |  |  | SusD-like protein P2 |
| 672 | CDS | 3195 |  |  |  | TonB-dependent receptor P3 |
| 673 | CDS | 1401 |  |  |  | hypothetical protein |
| 674 | CDS | 1203 |  |  |  | hypothetical protein |
| 675 | CDS | 420 |  |  |  | hypothetical protein |
| 676 | CDS | 348 |  |  |  | hypothetical protein |
| 677 | CDS | 2910 |  |  |  | hypothetical protein |
| 678 | CDS | 1536 |  | 3.1.6.- | COG3119 | N-acetylgalactosamine-6-O-sulfatase |
| 679 | CDS | 1224 | chuR | 1.1.99.- | COG0641 | Anaerobic sulfatase-maturating enzyme |
| 680 | CDS | 264 |  |  |  | hypothetical protein |
| 681 | CDS | 579 |  |  |  | hypothetical protein |
| 682 | CDS | 1377 | alsT |  | COG1115 | Amino-acid carrier protein AlsT |
| 683 | CDS | 582 |  |  |  | hypothetical protein |
| 684 | CDS | 684 |  |  |  | hypothetical protein |
| 685 | CDS | 477 | ribH | 2.5.1.78 |  | 6,7-dimethyl-8-ribityllumazine synthase |
| 686 | CDS | 615 |  |  |  | hypothetical protein |
| 687 | CDS | 540 | ygfA | 6.3.3.2 | COG0212 | 5-formyltetrahydrofolate cyclo-ligase |
| 688 | CDS | 450 | tadA | 3.5.4.33 |  | tRNA-specific adenosine deaminase |
| 689 | CDS | 675 |  |  |  | hypothetical protein |
| 690 | CDS | 1017 | apbE | 2.7.1.180 | COG1477 | FAD:protein FMN transferase |
| 691 | CDS | 774 |  |  |  | hypothetical protein |
| 692 | CDS | 465 |  |  |  | hypothetical protein |
| 693 | CDS | 633 |  |  |  | hypothetical protein |
| 694 | tRNA | 75 |  |  |  | tRNA-Thr(tgt) |
| 695 | CDS | 669 | thiE_2 | 2.5.1.3 |  | Thiamine-phosphate synthase |
| 696 | CDS | 879 | moaA_2 |  |  | GTP 3',8-cyclase |
| 697 | CDS | 825 | thiD | 2.7.1.49 | COG0351 | Hydroxymethylpyrimidine/phosphomethylpyrimidine kinase |
| 698 | CDS | 1095 | thiL | 2.7.4.16 | COG0611 | Thiamine-monophosphate kinase |
| 699 | CDS | 591 | punA | 2.4.2.1 | COG0005 | Purine nucleoside phosphorylase 1 |
| 700 | CDS | 858 | axeA1 | 3.1.1.72 | COG0657 | Acetylxylan esterase |
| 701 | CDS | 255 |  |  |  | hypothetical protein |
| 702 | CDS | 432 | mscL |  | COG1970 | Large-conductance mechanosensitive channel |
| 703 | CDS | 933 | ytlR | 2.7.1.- | COG1597 | Putative lipid kinase YtlR |
| 704 | CDS | 705 | crp |  | COG0664 | CRP-like cAMP-activated global transcriptional regulator |
| 705 | CDS | 597 | algU |  | COG1595 | RNA polymerase sigma-H factor |
| 706 | CDS | 657 | rpe | 5.1.3.1 |  | Ribulose-phosphate 3-epimerase |
| 707 | CDS | 810 | trpC | 4.1.1.48 |  | Indole-3-glycerol phosphate synthase |
| 708 | CDS | 630 | trpF | 5.3.1.24 | COG0135 | N-(5'-phosphoribosyl)anthranilate isomerase |
| 709 | CDS | 564 | pabA | 2.6.1.85 | COG0512 | Aminodeoxychorismate synthase component 2 |
| 710 | CDS | 978 | glcK | 2.7.1.2 | COG1940 | Glucokinase |
| 711 | CDS | 678 |  |  |  | hypothetical protein |
| 712 | CDS | 555 |  |  |  | hypothetical protein |
| 713 | CDS | 1809 | clcB |  |  | Voltage-gated ClC-type chloride channel ClcB |
| 714 | CDS | 573 | tsaC | 2.7.7.87 | COG0009 | Threonylcarbamoyl-AMP synthase |
| 715 | CDS | 615 |  |  |  | hypothetical protein |
| 716 | CDS | 513 |  |  |  | hypothetical protein |
| 717 | CDS | 1113 | yhcG_4 | 3.1.-.- | COG4804 | Putative nuclease YhcG |
| 718 | CDS | 1932 |  |  |  | hypothetical protein |
| 719 | CDS | 495 |  |  |  | hypothetical protein |
| 720 | CDS | 246 |  |  |  | hypothetical protein |
| 721 | CDS | 459 |  |  |  | hypothetical protein |
| 722 | CDS | 1275 | queA | 2.4.99.17 | COG0809 | S-adenosylmethionine:tRNA ribosyltransferase-isomerase |
| 723 | CDS | 705 |  |  |  | hypothetical protein |
| 724 | CDS | 750 |  |  |  | hypothetical protein |
| 725 | CDS | 1041 |  |  |  | hypothetical protein |
| 726 | CDS | 1221 |  |  |  | hypothetical protein |
| 727 | CDS | 819 | panB | 2.1.2.11 | COG0413 | 3-methyl-2-oxobutanoate hydroxymethyltransferase |
| 728 | CDS | 462 |  |  |  | hypothetical protein |
| 729 | CDS | 954 |  |  |  | hypothetical protein |
| 730 | CDS | 222 |  |  |  | hypothetical protein |
| 731 | CDS | 903 |  |  |  | hypothetical protein |
| 732 | CDS | 861 | ispE | 2.7.1.148 | COG1947 | 4-diphosphocytidyl-2-C-methyl-D-erythritol kinase |
| 733 | CDS | 2076 |  |  |  | hypothetical protein |
| 734 | CDS | 810 |  |  |  | hypothetical protein |
| 735 | CDS | 1284 |  |  |  | hypothetical protein |
| 736 | CDS | 555 |  |  |  | hypothetical protein |
| 737 | CDS | 561 |  |  |  | hypothetical protein |
| 738 | CDS | 480 | ispF | 4.6.1.12 | COG0245 | 2-C-methyl-D-erythritol 2,4-cyclodiphosphate synthase |
| 739 | CDS | 777 |  | 2.4.1.- |  | PGL/p-HBAD biosynthesis glycosyltransferase |
| 740 | CDS | 1446 |  |  |  | hypothetical protein |
| 741 | CDS | 960 |  |  |  | hypothetical protein |
| 742 | CDS | 720 | bcs1 |  |  | Bifunctional ribulose 5-phosphate reductase/CDP-ribitol pyrophosphorylase Bcs1 |
| 743 | CDS | 1029 | rfbB | 4.2.1.46 | COG1088 | dTDP-glucose 4,6-dehydratase |
| 744 | CDS | 816 |  |  |  | hypothetical protein |
| 745 | CDS | 1446 |  |  |  | hypothetical protein |
| 746 | CDS | 1026 |  |  |  | hypothetical protein |
| 747 | CDS | 1509 |  |  |  | hypothetical protein |
| 748 | CDS | 642 |  |  |  | hypothetical protein |
| 749 | CDS | 576 | gmk | 2.7.4.8 | COG0194 | Guanylate kinase |
| 750 | CDS | 606 |  |  |  | hypothetical protein |
| 751 | CDS | 522 | rimM |  |  | Ribosome maturation factor RimM |
| 752 | CDS | 354 |  |  |  | hypothetical protein |
| 753 | CDS | 2028 |  |  |  | hypothetical protein |
| 754 | CDS | 441 |  |  |  | hypothetical protein |
| 755 | CDS | 669 | truC | 5.4.99.26 | COG0564 | tRNA pseudouridine synthase C |
| 756 | CDS | 2400 | bamA |  |  | Outer membrane protein assembly factor BamA |
| 757 | CDS | 711 |  |  |  | hypothetical protein |
| 758 | CDS | 744 |  |  |  | hypothetical protein |
| 759 | CDS | 453 |  |  |  | hypothetical protein |
| 760 | CDS | 1965 |  |  |  | hypothetical protein |
| 761 | CDS | 1347 | mepA |  |  | Multidrug export protein MepA |
| 762 | CDS | 1152 | yghO_2 |  |  | Protein YghO |
| 763 | CDS | 1635 |  |  |  | hypothetical protein |
| 764 | CDS | 915 |  |  |  | hypothetical protein |
| 765 | CDS | 1059 |  |  |  | hypothetical protein |
| 766 | CDS | 855 | gph_2 | 3.1.3.18 |  | Phosphoglycolate phosphatase |
| 767 | CDS | 639 | exaE_2 |  |  | Transcriptional activator protein ExaE |
| 768 | CDS | 4155 | rcsC_4 | 2.7.13.3 |  | Sensor histidine kinase RcsC |
| 769 | CDS | 387 |  |  |  | hypothetical protein |
| 770 | CDS | 375 |  |  |  | hypothetical protein |
| 771 | CDS | 282 |  |  |  | hypothetical protein |
| 772 | CDS | 360 |  |  |  | hypothetical protein |
| 773 | CDS | 414 |  |  |  | hypothetical protein |
| 774 | CDS | 405 |  |  |  | hypothetical protein |
| 775 | CDS | 1563 |  |  |  | hypothetical protein |
| 776 | CDS | 432 |  |  |  | hypothetical protein |
| 777 | CDS | 729 |  |  |  | hypothetical protein |
| 778 | CDS | 2607 | hrpB | 3.6.4.13 | COG1643 | ATP-dependent RNA helicase HrpB |
| 779 | CDS | 1329 |  |  |  | hypothetical protein |
| 780 | CDS | 2478 | lon | 3.4.21.53 | COG0466 | Lon protease |
| 781 | CDS | 723 | yfiC | 2.1.1.223 | COG4123 | tRNA1(Val) (adenine(37)-N6)-methyltransferase |
| 782 | CDS | 360 | lrgA |  | COG1380 | Antiholin-like protein LrgA |
| 783 | CDS | 693 | lrgB |  | COG1346 | Antiholin-like protein LrgB |
| 784 | CDS | 1440 |  |  |  | hypothetical protein |
| 785 | CDS | 483 | cdd | 3.5.4.5 | COG0295 | Cytidine deaminase |
| 786 | CDS | 924 |  |  |  | hypothetical protein |
| 787 | CDS | 390 |  |  |  | hypothetical protein |
| 788 | CDS | 516 | cutD | 1.97.1.- |  | Choline trimethylamine-lyase activating enzyme |
| 789 | CDS | 2223 | nrdD | 1.1.98.6 | COG1328 | Anaerobic ribonucleoside-triphosphate reductase |
| 790 | CDS | 417 |  |  |  | hypothetical protein |
| 791 | CDS | 741 |  |  |  | hypothetical protein |
| 792 | CDS | 1368 |  |  |  | hypothetical protein |
| 793 | CDS | 408 |  |  |  | hypothetical protein |
| 794 | CDS | 435 |  |  |  | hypothetical protein |
| 795 | CDS | 864 | sigA_2 |  | COG0568 | RNA polymerase sigma factor SigA |
| 796 | CDS | 630 |  |  |  | hypothetical protein |
| 797 | CDS | 1026 |  |  |  | hypothetical protein |
| 798 | CDS | 2226 |  |  |  | hypothetical protein |
| 799 | CDS | 513 | rutE | 1.1.1.298 |  | malonic semialdehyde reductase RutE |
| 800 | CDS | 1095 |  |  |  | hypothetical protein |
| 801 | CDS | 2154 | btuB_2 |  |  | Vitamin B12 transporter BtuB |
| 802 | CDS | 1389 | umuC |  | COG0389 | Protein UmuC |
| 803 | CDS | 1164 |  |  |  | hypothetical protein |
| 804 | CDS | 477 |  |  |  | hypothetical protein |
| 805 | CDS | 1377 |  |  |  | hypothetical protein |
| 806 | CDS | 555 |  |  |  | hypothetical protein |
| 807 | CDS | 831 | rnz | 3.1.26.11 | COG1234 | Ribonuclease Z |
| 808 | CDS | 561 |  |  |  | hypothetical protein |
| 809 | CDS | 1803 |  |  |  | hypothetical protein |
| 810 | CDS | 873 |  |  |  | hypothetical protein |
| 811 | CDS | 822 |  |  |  | hypothetical protein |
| 812 | CDS | 495 |  |  |  | hypothetical protein |
| 813 | CDS | 558 |  |  |  | hypothetical protein |
| 814 | CDS | 306 |  |  |  | hypothetical protein |
| 815 | CDS | 741 |  |  |  | hypothetical protein |
| 816 | CDS | 246 |  |  |  | hypothetical protein |
| 817 | CDS | 276 |  |  |  | hypothetical protein |
| 818 | CDS | 468 |  |  |  | hypothetical protein |
| 819 | CDS | 243 |  |  |  | hypothetical protein |
| 820 | CDS | 615 |  |  |  | hypothetical protein |
| 821 | CDS | 426 |  |  |  | hypothetical protein |
| 822 | CDS | 480 |  |  |  | hypothetical protein |
| 823 | CDS | 252 |  |  |  | hypothetical protein |
| 824 | CDS | 813 |  |  |  | hypothetical protein |
| 825 | CDS | 363 |  |  |  | hypothetical protein |
| 826 | CDS | 921 | dinG | 3.1.-.- |  | 3'-5' exonuclease DinG |
| 827 | CDS | 972 |  |  |  | hypothetical protein |
| 828 | CDS | 465 |  |  |  | hypothetical protein |
| 829 | CDS | 255 |  |  |  | hypothetical protein |
| 830 | CDS | 441 |  |  |  | hypothetical protein |
| 831 | CDS | 1170 | sasA_3 | 2.7.-.- |  | Adaptive-response sensory-kinase SasA |
| 832 | CDS | 882 | era |  |  | GTPase Era |
| 833 | CDS | 378 |  |  |  | hypothetical protein |
| 834 | CDS | 768 | mkl |  | COG1127 | putative ribonucleotide transport ATP-binding protein mkl |
| 835 | CDS | 807 | lptB | 3.6.3.- | COG1137 | Lipopolysaccharide export system ATP-binding protein LptB |
| 836 | CDS | 1431 | prsA | 5.2.1.8 |  | Foldase protein PrsA |
| 837 | CDS | 1830 | lptD |  |  | LPS-assembly protein LptD |
| 838 | CDS | 885 |  |  |  | hypothetical protein |
| 839 | tRNA | 76 |  |  |  | tRNA-Val(tac) |
| 840 | tRNA | 76 |  |  |  | tRNA-Val(tac) |
| 841 | tRNA | 78 |  |  |  | tRNA-Val(tac) |
| 842 | CDS | 2010 |  |  |  | hypothetical protein |
| 843 | CDS | 204 |  |  |  | hypothetical protein |
| 844 | CDS | 639 |  |  |  | hypothetical protein |
| 845 | CDS | 663 |  |  |  | hypothetical protein |
| 846 | CDS | 426 |  |  |  | hypothetical protein |
| 847 | CDS | 951 |  |  |  | hypothetical protein |
| 848 | CDS | 750 | kdsA | 2.5.1.55 | COG2877 | 2-dehydro-3-deoxyphosphooctonate aldolase |
| 849 | CDS | 609 |  |  |  | hypothetical protein |
| 850 | CDS | 1227 |  |  |  | hypothetical protein |
| 851 | CDS | 1734 |  |  |  | hypothetical protein |
| 852 | CDS | 993 |  |  |  | hypothetical protein |
| 853 | CDS | 528 | dnrN |  | COG2846 | Iron-sulfur cluster repair protein DnrN |
| 854 | CDS | 957 |  |  |  | hypothetical protein |
| 855 | CDS | 492 |  |  |  | hypothetical protein |
| 856 | CDS | 336 |  |  |  | hypothetical protein |
| 857 | CDS | 2391 |  |  |  | hypothetical protein |
| 858 | CDS | 351 |  |  |  | hypothetical protein |
| 859 | CDS | 900 |  |  |  | hypothetical protein |
| 860 | CDS | 2034 |  |  |  | hypothetical protein |
| 861 | CDS | 465 |  |  |  | hypothetical protein |
| 862 | CDS | 1908 |  |  |  | hypothetical protein |
| 863 | CDS | 3138 | susC_3 |  |  | TonB-dependent receptor SusC |
| 864 | CDS | 1092 | rfbM | 2.7.7.13 | COG0662 | Mannose-1-phosphate guanylyltransferase RfbM |
| 865 | CDS | 1212 | fucP_3 |  | COG0738 | L-fucose-proton symporter |
| 866 | CDS | 876 |  |  |  | hypothetical protein |
| 867 | CDS | 1491 |  |  |  | hypothetical protein |
| 868 | CDS | 528 |  |  |  | hypothetical protein |
| 869 | CDS | 846 |  |  |  | hypothetical protein |
| 870 | CDS | 1551 |  |  |  | hypothetical protein |
| 871 | CDS | 1011 | xerC_3 |  |  | Tyrosine recombinase XerC |
| 872 | CDS | 498 |  |  |  | hypothetical protein |
| 873 | CDS | 342 |  |  |  | hypothetical protein |
| 874 | CDS | 3867 |  |  |  | hypothetical protein |
| 875 | CDS | 699 |  |  |  | hypothetical protein |
| 876 | CDS | 183 |  |  |  | hypothetical protein |
| 877 | CDS | 855 |  |  |  | hypothetical protein |
| 878 | CDS | 609 |  |  |  | hypothetical protein |
| 879 | CDS | 588 |  |  |  | hypothetical protein |
| 880 | CDS | 435 |  |  |  | hypothetical protein |
| 881 | CDS | 936 |  |  |  | hypothetical protein |
| 882 | CDS | 819 | pglE | 2.6.1.34 | COG0399 | UDP-N-acetylbacillosamine transaminase |
| 883 | CDS | 267 |  |  |  | hypothetical protein |
| 884 | CDS | 1845 |  |  |  | hypothetical protein |
| 885 | CDS | 831 |  |  |  | hypothetical protein |
| 886 | CDS | 1221 |  |  |  | hypothetical protein |
| 887 | CDS | 591 | wecA_2 | 2.7.8.40 |  | UDP-N-acetylgalactosamine-undecaprenyl-phosphate N-acetylgalactosaminephosphotransferase |
| 888 | CDS | 1290 | arnB | 2.6.1.87 | COG0399 | UDP-4-amino-4-deoxy-L-arabinose--oxoglutarate aminotransferase |
| 889 | CDS | 795 | wbbD | 2.4.1.303 | COG0463 | UDP-Gal:alpha-D-GlcNAc-diphosphoundecaprenol beta-1,3-galactosyltransferase |
| 890 | CDS | 882 |  |  |  | hypothetical protein |
| 891 | CDS | 420 | tarD | 2.7.7.39 | COG0615 | Glycerol-3-phosphate cytidylyltransferase |
| 892 | CDS | 924 |  |  |  | hypothetical protein |
| 893 | CDS | 840 | mshA_1 | 2.4.1.250 |  | D-inositol-3-phosphate glycosyltransferase |
| 894 | CDS | 711 |  |  |  | hypothetical protein |
| 895 | CDS | 846 |  |  |  | hypothetical protein |
| 896 | CDS | 810 |  |  |  | hypothetical protein |
| 897 | CDS | 1137 |  |  |  | hypothetical protein |
| 898 | CDS | 1428 | rfbX |  | COG2244 | Putative O-antigen transporter |
| 899 | CDS | 1419 |  |  |  | hypothetical protein |
| 900 | CDS | 909 | rmlD | 1.1.1.133 | COG1091 | dTDP-4-dehydrorhamnose reductase |
| 901 | CDS | 909 | rffH | 2.7.7.24 | COG1209 | Glucose-1-phosphate thymidylyltransferase 2 |
| 902 | CDS | 303 |  |  |  | hypothetical protein |
| 903 | CDS | 294 |  |  |  | hypothetical protein |
| 904 | CDS | 1137 |  |  |  | hypothetical protein |
| 905 | CDS | 2286 |  |  |  | hypothetical protein |
| 906 | CDS | 1755 |  |  |  | hypothetical protein |
| 907 | CDS | 2283 |  |  |  | hypothetical protein |
| 908 | CDS | 1734 |  |  |  | hypothetical protein |
| 909 | CDS | 801 | fhuC | 7.2.2.16 | COG1120 | Iron(3+)-hydroxamate import ATP-binding protein FhuC |
| 910 | CDS | 825 | yidA | 3.1.3.23 | COG0561 | Sugar phosphatase YidA |
| 911 | CDS | 501 |  |  |  | hypothetical protein |
| 912 | CDS | 774 |  |  |  | hypothetical protein |
| 913 | CDS | 909 |  |  |  | hypothetical protein |
| 914 | CDS | 1551 |  |  |  | SusD-like protein P38 |
| 915 | CDS | 3057 |  |  |  | TonB-dependent receptor P3 |
| 916 | CDS | 342 |  |  |  | hypothetical protein |
| 917 | CDS | 1374 |  |  |  | hypothetical protein |
| 918 | CDS | 2412 |  |  |  | hypothetical protein |
| 919 | CDS | 2034 |  |  |  | hypothetical protein |
| 920 | CDS | 768 | cheB_2 | 3.5.1.44 |  | Protein-glutamate methylesterase/protein-glutamine glutaminase |
| 921 | CDS | 1233 |  |  |  | hypothetical protein |
| 922 | CDS | 804 |  |  |  | hypothetical protein |
| 923 | CDS | 660 |  |  |  | hypothetical protein |
| 924 | CDS | 1269 |  |  |  | hypothetical protein |
| 925 | CDS | 2472 | etk | 2.7.10.- | COG0489 | Tyrosine-protein kinase etk |
| 926 | CDS | 651 |  |  |  | hypothetical protein |
| 927 | CDS | 291 |  |  |  | hypothetical protein |
| 928 | CDS | 984 | xerC_4 |  |  | Tyrosine recombinase XerC |
| 929 | CDS | 1341 |  |  |  | hypothetical protein |
| 930 | CDS | 966 |  |  |  | hypothetical protein |
| 931 | CDS | 1140 |  |  |  | hypothetical protein |
| 932 | CDS | 2793 |  |  |  | hypothetical protein |
| 933 | CDS | 426 |  |  |  | hypothetical protein |
| 934 | CDS | 594 |  |  |  | hypothetical protein |
| 935 | CDS | 1155 |  |  |  | hypothetical protein |
| 936 | CDS | 1662 |  |  |  | hypothetical protein |
| 937 | CDS | 1128 |  |  |  | hypothetical protein |
| 938 | CDS | 1047 |  |  |  | hypothetical protein |
| 939 | CDS | 1926 |  |  |  | hypothetical protein |
| 940 | CDS | 1728 |  |  |  | hypothetical protein |
| 941 | CDS | 1479 |  |  |  | hypothetical protein |
| 942 | CDS | 1080 | macA_2 |  | COG0845 | Macrolide export protein MacA |
| 943 | CDS | 3081 | mdtC_2 |  |  | Multidrug resistance protein MdtC |
| 944 | CDS | 3180 | ttgH |  |  | Toluene efflux pump membrane transporter TtgH |
| 945 | CDS | 624 |  |  |  | hypothetical protein |
| 946 | CDS | 537 |  |  |  | hypothetical protein |
| 947 | CDS | 1080 |  |  |  | hypothetical protein |
| 948 | CDS | 1011 |  |  |  | hypothetical protein |
| 949 | CDS | 696 | walR |  | COG0745 | Transcriptional regulatory protein WalR |
| 950 | CDS | 1461 | sasA_4 | 2.7.-.- |  | Adaptive-response sensory-kinase SasA |
| 951 | tRNA | 75 |  |  |  | tRNA-Asn(gtt) |
| 952 | tRNA | 75 |  |  |  | tRNA-Asn(gtt) |
| 953 | CDS | 1179 | resA_3 |  |  | Thiol-disulfide oxidoreductase ResA |
| 954 | CDS | 534 | nudC | 3.6.1.22 |  | NADH pyrophosphatase |
| 955 | CDS | 528 | aroK | 2.7.1.71 | COG0703 | Shikimate kinase |
| 956 | CDS | 882 |  |  |  | hypothetical protein |
| 957 | CDS | 1041 | mshA_2 | 2.4.1.250 |  | D-inositol-3-phosphate glycosyltransferase |
| 958 | CDS | 1233 | mshA_3 | 2.4.1.250 |  | D-inositol-3-phosphate glycosyltransferase |
| 959 | CDS | 2013 |  |  |  | hypothetical protein |
| 960 | CDS | 1011 |  |  |  | hypothetical protein |
| 961 | CDS | 1137 |  |  |  | hypothetical protein |
| 962 | CDS | 3447 |  |  |  | hypothetical protein |
| 963 | CDS | 1344 |  |  |  | hypothetical protein |
| 964 | CDS | 714 | tagA | 2.4.1.187 | COG1922 | N-acetylglucosaminyldiphosphoundecaprenol N-acetyl-beta-D-mannosaminyltransferase |
| 965 | CDS | 810 | soj_2 | 3.6.-.- | COG1192 | Sporulation initiation inhibitor protein Soj |
| 966 | CDS | 504 |  |  |  | hypothetical protein |
| 967 | CDS | 303 |  |  |  | hypothetical protein |
| 968 | CDS | 1938 |  |  |  | hypothetical protein |
| 969 | CDS | 975 |  |  |  | hypothetical protein |
| 970 | CDS | 966 |  |  |  | hypothetical protein |
| 971 | CDS | 984 |  |  |  | hypothetical protein |
| 972 | CDS | 267 |  |  |  | hypothetical protein |
| 973 | CDS | 294 |  |  |  | hypothetical protein |
| 974 | CDS | 351 |  |  |  | hypothetical protein |
| 975 | CDS | 414 | yrrK | 3.1.-.- | COG0816 | Putative pre-16S rRNA nuclease |
| 976 | CDS | 561 | def | 3.5.1.88 | COG0242 | Peptide deformylase |
| 977 | CDS | 1032 | ansA | 3.5.1.1 | COG0252 | L-asparaginase 1 |
| 978 | CDS | 618 |  |  |  | hypothetical protein |
| 979 | CDS | 465 |  |  |  | hypothetical protein |
| 980 | CDS | 432 |  |  |  | hypothetical protein |
| 981 | CDS | 1590 |  |  |  | hypothetical protein |
| 982 | CDS | 711 |  |  |  | hypothetical protein |
| 983 | CDS | 546 |  |  |  | hypothetical protein |
| 984 | CDS | 1431 |  |  |  | hypothetical protein |
| 985 | CDS | 756 |  |  |  | hypothetical protein |
| 986 | CDS | 243 |  |  |  | hypothetical protein |
| 987 | CDS | 969 |  |  |  | hypothetical protein |
| 988 | CDS | 522 |  |  |  | hypothetical protein |
| 989 | CDS | 735 |  |  |  | hypothetical protein |
| 990 | CDS | 444 |  |  |  | hypothetical protein |
| 991 | CDS | 1578 |  |  |  | hypothetical protein |
| 992 | CDS | 369 |  |  |  | hypothetical protein |
| 993 | CDS | 582 |  |  |  | hypothetical protein |
| 994 | CDS | 852 |  |  |  | hypothetical protein |
| 995 | CDS | 780 |  |  |  | hypothetical protein |
| 996 | CDS | 612 |  |  |  | hypothetical protein |
| 997 | CDS | 480 |  |  |  | hypothetical protein |
| 998 | CDS | 801 |  |  |  | hypothetical protein |
| 999 | CDS | 480 |  |  |  | hypothetical protein |
| 1000 | CDS | 702 |  |  |  | hypothetical protein |
| 1001 | CDS | 417 |  |  |  | hypothetical protein |
| 1002 | CDS | 762 |  |  |  | hypothetical protein |
| 1003 | CDS | 1686 |  |  |  | hypothetical protein |
| 1004 | CDS | 600 | fabG | 1.1.1.100 |  | 3-oxoacyl-[acyl-carrier-protein] reductase FabG |
| 1005 | CDS | 1812 | ltrA |  | COG3344 | Group II intron-encoded protein LtrA |
| 1006 | CDS | 570 |  |  |  | hypothetical protein |
| 1007 | CDS | 1215 |  |  |  | hypothetical protein |
| 1008 | CDS | 2004 |  |  |  | hypothetical protein |
| 1009 | CDS | 450 |  |  |  | hypothetical protein |
| 1010 | CDS | 1083 |  |  |  | hypothetical protein |
| 1011 | CDS | 441 |  |  |  | hypothetical protein |
| 1012 | CDS | 1275 |  |  |  | hypothetical protein |
| 1013 | CDS | 780 |  |  |  | hypothetical protein |
| 1014 | CDS | 642 |  |  |  | hypothetical protein |
| 1015 | CDS | 663 |  |  |  | hypothetical protein |
| 1016 | CDS | 789 |  |  |  | hypothetical protein |
| 1017 | CDS | 798 |  |  |  | hypothetical protein |
| 1018 | CDS | 1005 |  |  |  | hypothetical protein |
| 1019 | CDS | 621 | pflA | 1.97.1.4 | COG1180 | Pyruvate formate-lyase 1-activating enzyme |
| 1020 | CDS | 1977 |  |  |  | hypothetical protein |
| 1021 | CDS | 1134 |  |  |  | Outer membrane protein 40 |
| 1022 | CDS | 1170 |  |  |  | hypothetical protein |
| 1023 | CDS | 1053 |  |  |  | hypothetical protein |
| 1024 | CDS | 411 |  |  |  | hypothetical protein |
| 1025 | CDS | 447 |  |  |  | hypothetical protein |
| 1026 | CDS | 1047 |  |  |  | hypothetical protein |
| 1027 | CDS | 570 |  |  |  | hypothetical protein |
| 1028 | CDS | 1188 |  |  |  | hypothetical protein |
| 1029 | CDS | 2739 |  |  |  | hypothetical protein |
| 1030 | CDS | 345 |  |  |  | hypothetical protein |
| 1031 | CDS | 624 |  |  |  | hypothetical protein |
| 1032 | CDS | 1293 |  |  |  | hypothetical protein |
| 1033 | CDS | 1341 |  |  |  | hypothetical protein |
| 1034 | CDS | 543 |  |  |  | hypothetical protein |
| 1035 | rRNA | 1316 |  |  |  | 16S ribosomal RNA |
| 1036 | rRNA | 999 |  |  |  | 23S ribosomal RNA (partial) |
| 1037 | tRNA | 75 |  |  |  | tRNA-Ile(gat) |
